# A Spatially Directed Microneedle Patch Enables Intratumoral Co‐Delivery of FOLFIRINOX, Surufatinib, and Anti‐PD‐1 for Chemo‐Immunotherapy of Pancreatic Ductal Adenocarcinoma

**DOI:** 10.1002/advs.76478

**Published:** 2026-07-13

**Authors:** Tingting Kong, Ximo Xu, Xiao Zhang, Chuntao Wu, Zhengjun Qiu, Beiyuan Hu, Zihao Qi, Qiang Tian, Yuqin Yang, Hanguang Dong, Fei Wu, Tuo Jin, Yan Zheng, Jiang Long

**Affiliations:** ^1^ Department of Pancreatic Surgery Shanghai Key Laboratory of Pancreatic Disease Shanghai General Hospital Shanghai Jiao Tong University School of Medicine Shanghai China; ^2^ Department of Gastroenterology Shanghai General Hospital Shanghai Jiao Tong University School of Medicine Shanghai China; ^3^ Department of Laboratory Animal Center Shanghai General Hospital Shanghai Jiao Tong University School of Medicine Shanghai China; ^4^ Shanghai Institute of Hematology State Key Laboratory of Medical Genomics National Research Center for Translational Medicine at Shanghai Ruijin Hospital Affiliated to Shanghai Jiao Tong University School of Medicine Shanghai China; ^5^ School of Pharmacy Shanghai Jiao Tong University Shanghai China

**Keywords:** chemo‐immunotherapy strategy, localized drug delivery, microneedle, pancreatic ductal adenocarcinoma, tumor microenvironment

## Abstract

Pancreatic ductal adenocarcinoma (PDAC), a lethal malignancy with a 5‐year survival of 13%, faces limited therapeutic efficacy due to fibrotic barriers that block drug access to the therapeutic site, systemic toxicity of conventional chemotherapy, and an immunosuppressive tumor microenvironment (TME). Therapeutic agents with different functions, including FOLFIRINOX (FFX), surufatinib (SUR), and anti‐PD‐1 (αPD‐1), are co‐administered to tumors in animal models using microneedle (MN) patches to achieve site‐targeted synergistic efficacy. The core–shell MN structure is used, with the outer layer providing rapid FFX delivery and the inner layer providing sustained release of SUR and αPD‐1. Compared with intratumoral drug injection and traditional systemic administration, MN patch‐mediated administration markedly suppresses tumor growth and liver metastasis at lower doses, reduces systemic drug exposure and toxicity, and prolongs intratumoral drug retention. In addition, the MN patch‐mediated intratumoral co‐delivery reshapes the TME by boosting CD8^+^ T cells infiltration, inhibiting Foxp3^+^ regulatory T cells, and reducing CD206^+^ tumor‐associated macrophages infiltration, resulting in downregulation of tumor epithelial‐mesenchymal transition and increased tumor cell apoptosis. In summary, MN patch‐mediated delivery of therapeutic agents provides sufficient intratumoral doses and minimizes systemic toxicity through multiple microchannels, suggesting that MN patch co‐delivery is a promising adjunct for enhancing efficacy against PDAC.

## Graphical abstract

A double‐layered shell‐core microneedle patch is developed to co‐deliver FOLFIRINOX, surufatinib, and anti‐PD‐1 for localized chemo‐immunotherapy of PDAC. This strategy achieves sustained tumor suppression, reduces metastasis, and reprograms the TME by enhancing CD8^+^ T‐cell infiltration and inhibiting Tregs and M2 macrophages infiltration, while minimizing systemic toxicity and offering a high‐efficacy, low‐toxicity therapeutic strategy for PDAC.

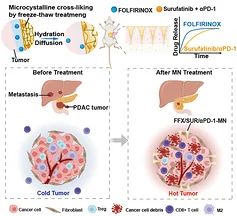

## 1 Introduction

Pancreatic ductal adenocarcinoma (PDAC) is projected to become the third leading cause of cancer‐related mortality, with a 5‐year overall survival (OS) rate remaining at only 13% [1]. The poor prognosis is attributed to late diagnosis, with more than 50% of cases presenting with systemic metastasis. Traditional fluorouracil‐ or gemcitabine‐based induction therapy, with or without radiation, remains the standard of care for patients with unresectable PDAC. However, this approach is associated with limited therapeutic efficacy, low rates of conversion to surgical resectability [2], and substantial systemic toxicities. Therefore, the development of more effective induction therapies remains a critical unmet clinical need.

The highly fibrotic stroma and immunosuppressive TME constitute fundamental barriers to effective drug delivery. Most of the PDAC tumor mass consists of rigid stroma, which suppresses cytotoxic T lymphocytes (CTLs) through C‐X‐C motif chemokine ligand 12 (CXCL12) and interleukin‐10 (IL‐10) secretion [3, 4, 5, 6, 7, 8, 9, 10]. In addition, *KRAS* mutations are prevalent in PDAC, driving granulocyte‐macrophages colony‐stimulating factor (GM‐CSF) release, recruiting myeloid‐derived suppressor cells (MDSCs), and exacerbating fibroinflammatory responses. Tumor cells evade immunity via autophagy‐mediated major histocompatibility complex class I (MHC‐I) degradation, CD47 overexpression, and indoleamine 2,3‐dioxygenase (IDO) activity [11, 12]. Recent clinical trials (NCT0521889) combining nab‐paclitaxel regimen with surufatinib (SUR), camrelizumab, and S‐1 (NASCA) showed improved objective response rate (ORR) (55.0% vs. 23.1%) and extended median progression‐free survival (8.8 vs. 5.8 months). Subgroup analysis highlighted enhanced ORR in liver metastasis patients (90.0% vs. 20.0%, *p =* 0.0017). However, the combination therapy showed obviously increased toxicity profiles, including hepatotoxicity and diarrhea [13]. While these findings underscore the therapeutic promise of the combination regimen, they simultaneously reveal its dose‐limiting toxicities. Conventional systemic administration faces limitations in dosage control, tissue penetration, and maintenance of therapeutic plasma concentrations, frequently leading to myelosuppression, gastrointestinal complications, and narrow therapeutic windows. FOLFIRINOX (FFX) is a combination of oxaliplatin (LOHP), irinotecan (CPT‐11), leucovorin (LV), and 5‐fluorouracil (5‐FU), which is a standard first‐line chemotherapy regimen for advanced PDAC [14]. Surufatinib, a multi‐targeted tyrosine kinase inhibitor (TKI) that targets fibroblast growth factor receptor 1 (FGFR1), colony‐stimulating factor 1 receptor (CSF1R), and vascular endothelial growth factor receptors 1–3 (VEGFR1‐3), may enhance the permeability of chemotherapy through extravasation barriers [15]. Meanwhile, the suboptimal response rates to anti‐PD‐1 monotherapy in PDAC highlight the necessity for localized remodeling of the immune microenvironment to enhance therapeutic efficacy [16, 17].

MN technology, leveraging its micron‐scale needle array architecture, enables localized drug enrichment, systemic toxicity mitigation, precision modulation of release kinetics, and enhanced macromolecular payload delivery. It has emerged as a precision oncology therapeutic [18, 19, 20, 21, 22, 23]. Compared to intratumoral injection, microneedle drug delivery, with its multiple administration sites, ensures more uniform drug distribution, better tumor suppression, and reduced toxic side effects. Here, we developed a chemo‐targeted‐immunotherapy (FFX/SUR/αPD‐1) approach with a double‐layered shell‐core MN system to achieve temporal drug delivery and high anticancer efficacy (Figure 1A–D). The outer layer, composed of rapidly degradable polyvinylpyrrolidone (PVP) and polyvinyl alcohol (PVA), was designed to load FFX for immediate drug release within 24 h post MN penetration into tumor tissue. The core layer, composed of PVA and encapsulating SUR and αPD‐1, is formed through physical crosslinking during freeze‐thaw cycles. This process avoids the use of organic reagents, thereby effectively preserving the activity of αPD‐1. Upon penetrating the tumor tissue, the core layer absorbs water and swells, creating microchannels within the MN that facilitate the sustained release of SUR and αPD‐1 over 7 days. This temporal delivery strategy suppresses tumor growth and liver metastasis via epithelial‐mesenchymal transition (EMT) inhibition and immune activation. Compared to conventional systemic administration approaches, our study provides a high‐efficacy, low‐toxicity induction therapy for unresectable PDAC.

**FIGURE 1:**
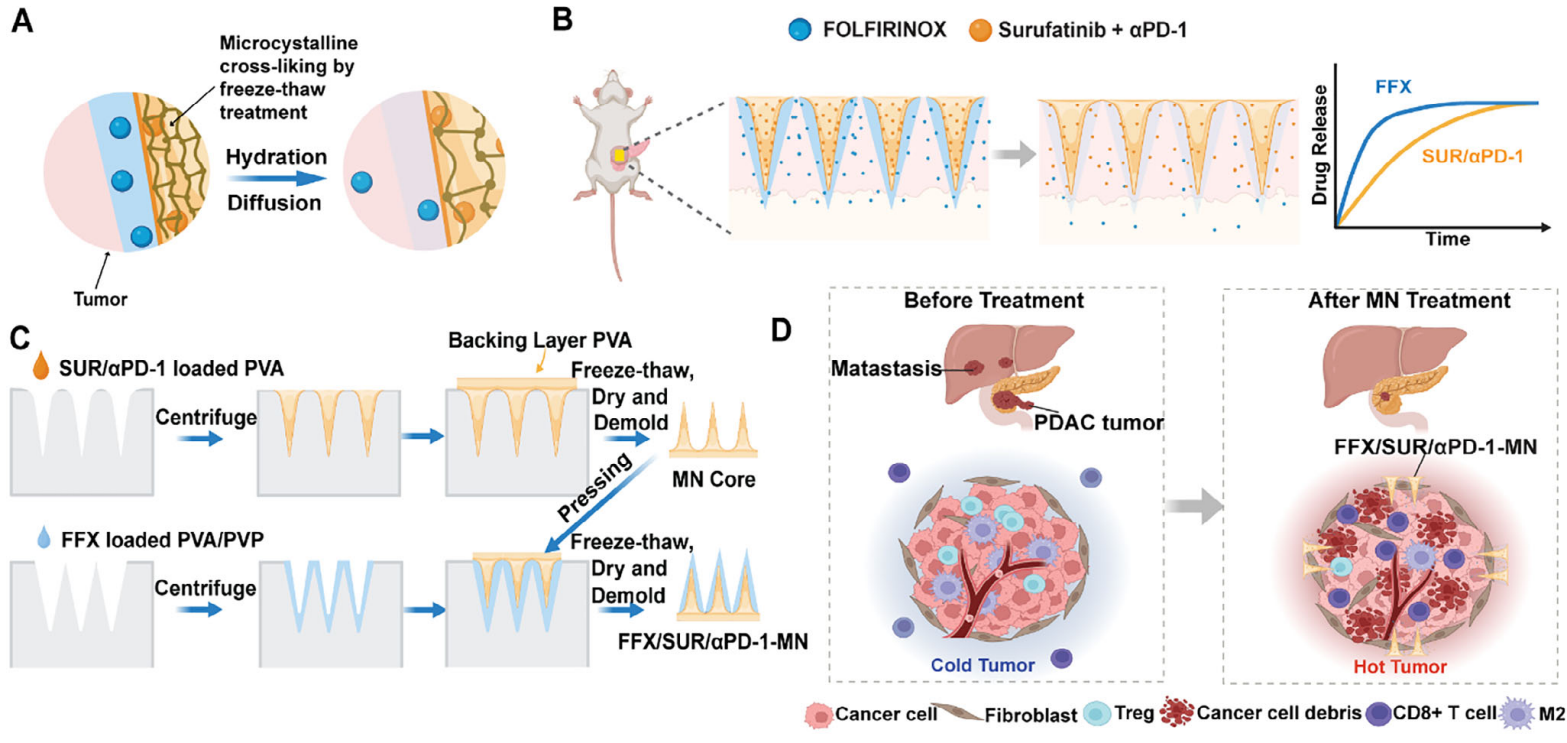
Schematic of the MN patch showing FFX rapid release, SUR/αPD‐1 sustained release, and modulation of the PDAC tumor microenvironment (Created with BioRender.com). (A) Mechanism of FFX/SUR/αPD‐1 release from the MN patch. Upon insertion into tumor tissue, interstitial fluid permeates the shell, dissolving the PVP within and enabling subsequent diffusion of FFX into the tumor microenvironment. The core, prepared via PVA crystalline cross‐linking, swells upon absorbing interstitial fluid, forming microchannels within the needle structure through which SUR and αPD‐1 are subsequently released into the tumor tissue. A higher number of freeze‐thaw cycles increases the density of crystalline cross‐linking points, resulting in narrower microchannels and slower drug release rates. (B) Action of the FFX/SUR/αPD‐1‐MN patch on orthotopic pancreatic cancer tumors in the mouse model. The outer layer enables rapid degradation and release of FFX, while the inner layer facilitates sustained release of SUR and αPD‐1. (C) Schematic of the preparation process for the FFX/SUR/αPD‐1‐MN patch. (D) The schematic illustration shows that MN treatment achieved sustained tumor suppression, reduced metastasis, and reprogrammed the TME by enhancing CD8^+^ T‐cell infiltration, inhibiting Tregs and M2 macrophages infiltration.

## 2 Results

### 2.1 FFX/SUR/αPD‐1 Therapy Significantly Inhibits PDAC Progression

The dual‐combination therapies of FFX plus αPD‐1 or SUR plus αPD‐1 have been reported in the literature, but the outcomes have not been satisfactory [24, 25, 26]. To address these resistance mechanisms of the highly fibrotic stroma and immunosuppressive microenvironment in PDAC, we proposed a FFX/SUR/αPD‐1 therapy. To evaluate its therapeutic potential on PDAC, we established orthotopic pancreatic tumors in C57BL/6 mice through surgical implantation of Panc02 cells (Figure 2A). The FFX/SUR/αPD‐1 therapy group showed markedly reduced primary tumor volume (day 28) and liver metastases compared to FFX plus αPD‐1 or SUR plus αPD‐1 groups (Figure 2B–H). Corresponding TUNEL and Ki67 staining further confirmed enhanced tumor apoptosis and reduced proliferation in the FFX/SUR/αPD‐1 group (Figure S1A–C). Continuous treatment with FFX/SUR/αPD‐1 improved overall survival compared to the control groups (Figure 2I). However, the FFX/SUR/αPD‐1 therapy group exhibited decreased body weight in mice, likely due to diarrhea, and also demonstrated more severe liver function impairment compared to the control and dual‐combination groups (Figure 2J–N). These findings indicate a remarkable synergistic effect of the FFX/SUR/αPD‐1 therapy on PDAC progression, while also demonstrating toxicity profiles.

**FIGURE 2:**
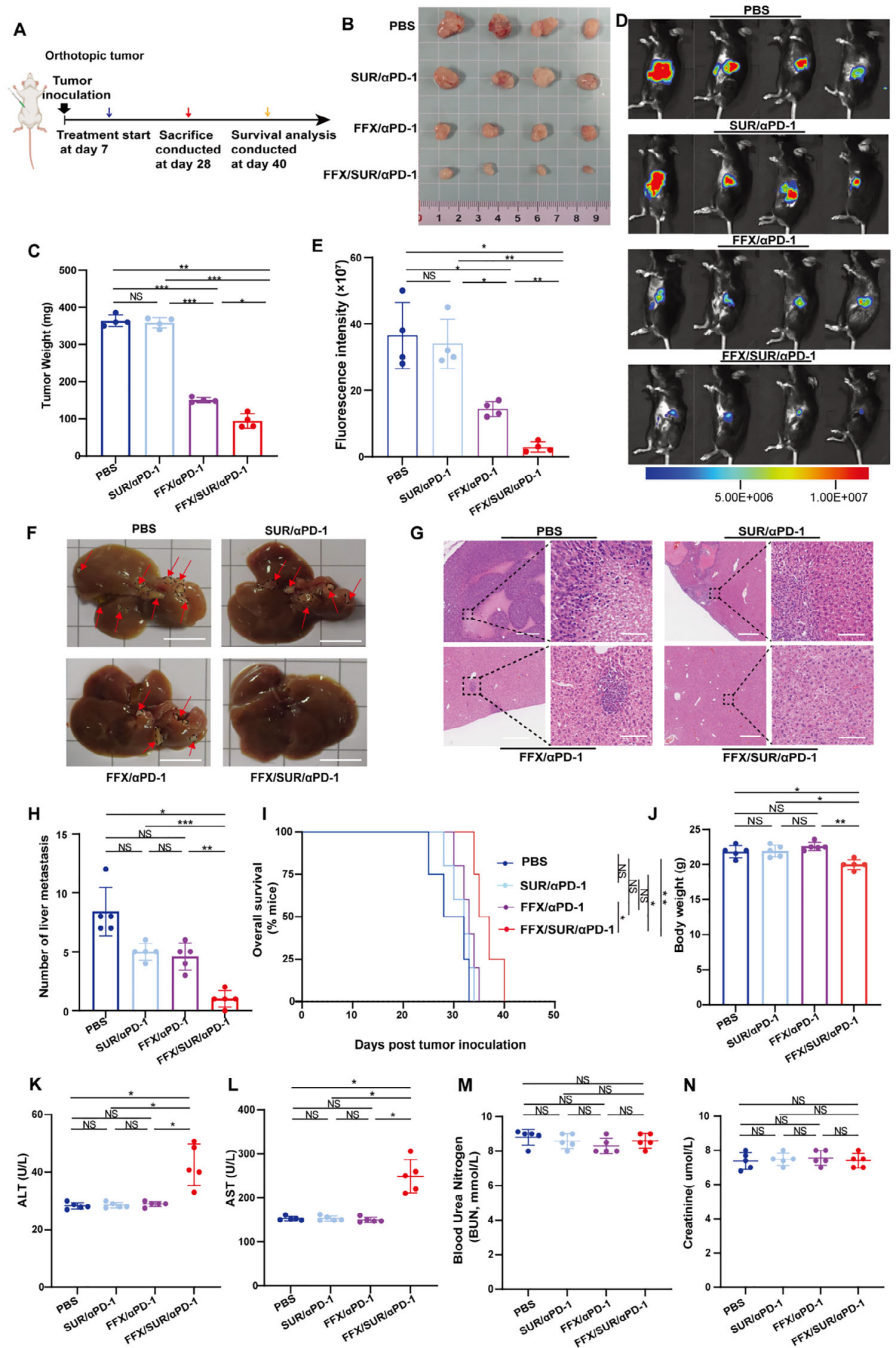
FFX/SUR/αPD‐1 therapy enhances anti‐tumor efficacy and suppresses liver metastasis in the PDAC mouse model. (A) C57BL/6 mice were orthotopically implanted with Panc02‐luc tumors. Schematic of the treatment protocol and survival monitoring schedule for the orthotopic PDAC mouse model (Created with BioRender.com). Five mice per group were treated with PBS, SUR (p.o.) plus αPD‐1 (i.p.), FFX (i.p.) plus αPD‐1 (i.p.), FFX (i.p.) plus SUR (p.o.) plus αPD‐1 (i.p.) until the treatment endpoint. (B–E) Tumor images (B), tumor weights (C), in vivo fluorescence imaging (D), and fluorescence intensity (E) of orthotopic Panc02 tumor‐bearing mice at 28 days after tumor inoculation, as described in panel A (*n* = 4 mice per group, biologically independent samples). (F–H) Representative images of liver tissues (scale bars, 10 mm) (F), H&E‐stained liver sections (red arrows indicate tumor lesions (scale bars, 500 µm [left], 100 µm [right]) (G), and the number of liver metastatic foci (H) from each group (*n* = 5 mice per group, biologically independent samples). (I) Kaplan–Meier survival curves of the mouse model, and the log‐rank test was performed (*n* = 10 mice per group, biologically independent samples). (J–N) The body weight (J), alanine aminotransferase (ALT) (K), aspartate aminotransferase (AST) (L), blood urea nitrogen (M), and creatinine (N) of each group at the time of sacrifice (*n* = 5 mice per group, biologically independent samples). Statistical analysis was performed using one‐way ANOVA (C, E, H, J and K–N) and log‐rank test (I). Data are presented as the mean ± SD. ^***^ *p* < 0.001, ^**^ *p* < 0.01, ^*^ *p* < 0.05; NS, not significant.

### 2.2 Preparation and Characterization of the FFX/SUR/αPD‐1‐MN Patch

The MN system comprised a SUR/αPD‐1‐loaded core and a FFX‐loaded shell. Initially, the core was fabricated by casting a solution containing PVA, SUR, and αPD‐1 into MN molds, followed by freeze‐thaw cycles three times and crystallization crosslinking. The shell, formulated with PVP, PVA, and FFX (5‐fluorouracil, leucovorin, irinotecan, and oxaliplatin), was cast into complementary molds. Core–shell integration was achieved through precise alignment of the prefabricated core with the shell mold, subsequent freeze‐thaw cycles, drying, and demolding (Figure 1B). PVP was selected as the major shell component because of its high water solubility and rapid dissolution upon contact with interstitial fluid, enabling the immediate release of FFX for initial tumor debulking. A small proportion of PVA was added to the shell to reinforce mechanical strength and improve compatibility with the PVA‐based core, ensuring robust core–shell integration. In contrast, the core was composed of PVA processed by repeated freeze‐thaw crystallization, which formed a semi‐crystalline structure with microchannels on the MN body. These microchannels allowed the sustained release of SUR and αPD‐1 into the tissue, while the crystallization process also helped preserve the bioactivity of αPD‐1.

The fabricated patch featured a 10 × 10 array of sharp MNs (0.5 cm^2^), with individual needles exhibiting a base diameter of 300 µm, a distance of 300 µm between the needles, and a height of 500 µm (Figure 3A,B). Scanning electron microscopy (SEM) confirmed the structural integrity, and fluorescence imaging validated the bilayer core–shell architecture (Figure 3B,C). Mechanical testing using a force gauge demonstrated a fracture force of ∼1.18 N per needle and a maximum pressure tolerance of 16.7 mPa (Figure 3D), sufficient to penetrate human PDAC tissue (∼6 kPa) [22, 27, 28, 29, 30, 31]. Quantitative analysis of loaded αPD‐1 in five different regions of MN patches fabricated in different batches also substantiated the uniform distribution and good reproducibility (Figure 3E). Furthermore, the fabrication process did not affect the bioactivity of αPD‐1 within the MNs, as evidenced by the negligible difference in bioactivity between freshly prepared MNs and native αPD‐1 (Figure 3F). Drug‐loading analysis showed that all agents were incorporated into the MN patches with > 95% encapsulation efficiency, with measured doses matching the designed loading amounts. The drug in MNs could well maintain its bioactivity at room temperature under dry conditions for at least 24 weeks (Figure 3G). In addition, the biosafety of the MN material was further validated by a cytotoxicity test (Figure 3H). The prepared MNs possess high drug‐loading capacity, uniform drug distribution, long‐term stability, and biocompatibility that meet the requirements for practical application.

**FIGURE 3:**
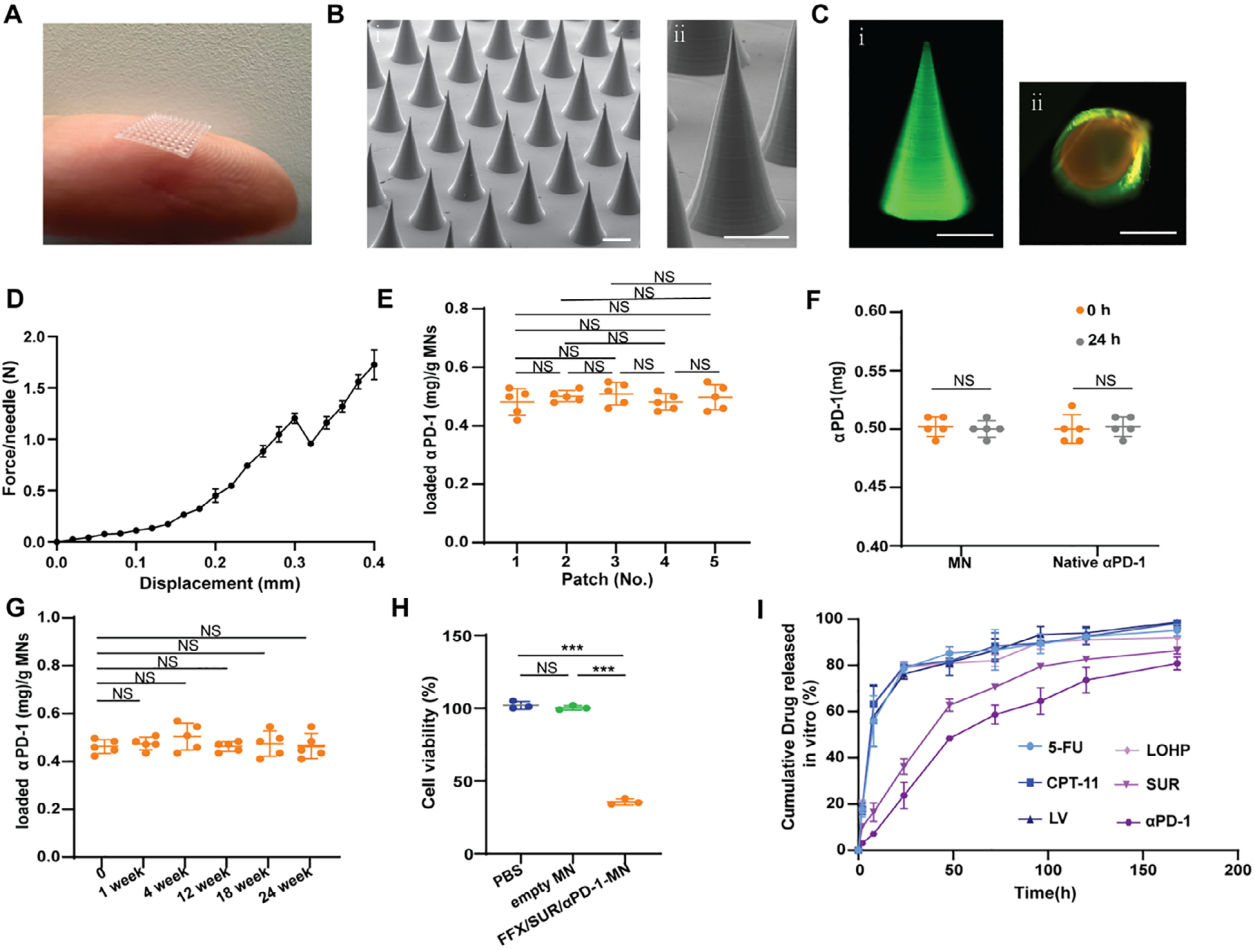
Preparation and characterization of the FFX/SUR/αPD‐1‐MN patch. (A) Photograph of the prepared FFX/SUR/αPD‐1‐MN patch. (B) Scanning electron microscopy (SEM) images of the FFX/SUR/αPD‐1‐MN array (i) and an enlarged single FFX/SUR/αPD‐1‐MN (ii) (scale bars, 200 µm). (C) Fluorescence images of a complete needle body (i), and cross‐sections of an MN patch showing a red core labeled with Nile Red and a green shell labeled with FITC (scale bars, 200 µm) (ii). (D) Mechanical behavior of the MN patch (*n* = 3 FFX/SUR/αPD‐1‐MN patches, biologically independent samples). (E) Amount of loaded αPD‐1 of nine MNs in five different regions from one FFX/SUR/αPD‐1‐MN patch. Patch (No.) represents FFX/SUR/αPD‐1‐MN patches produced in five different batches (*n* = 5 regions/FFX/SUR/αPD‐1‐MN patch, biologically independent samples). (F) Bioactivity comparison between the native αPD‐1 and αPD‐1 extracted from FFX/SUR/αPD‐1‐MN (*n* = 5 FFX/SUR/αPD‐1‐MN patches, biologically independent samples). (G) Long‐term bioactivity of αPD‐1 extracted from FFX/SUR/αPD‐1‐MN patches stored at room temperature under a dry condition (*n* = 5 FFX/SUR/αPD‐1‐MN patches, biologically independent samples). (H) Statistical analysis of relative Panc02 cells viability after PBS, empty MN, and FFX/SUR/αPD‐1‐MN treatment at 6 h (*n* = 3, biologically independent samples). (I) Drug release characterization of the FFX/SUR/αPD‐1‐MN patches in PBS solution at 37°C. Date are shown as cumulative release over time (*n* = 5, biologically independent samples). Statistical analysis was performed using one‐way ANOVA (E and H) and unpaired two‐tailed Student's *t*‐test (F and G). Data are presented as the mean ± SD. ^***^ *p* < 0.001, ^**^ *p* < 0.01, ^*^ *p* < 0.05; NS, not significant.

The MN patches were subjected to in vitro drug release profiling in phosphate‐buffered saline (PBS, pH 7.4) at 37°C. Drug release kinetics revealed that FFX achieved rapid release within 24 h, while SUR and αPD‐1 exhibited essentially no burst release and sustained release profiles at a rate of ∼12% per day over 7 days (Figure 3I). These differential release patterns align with therapeutic design objectives.

### 2.3 Low‐Dose FFX/SUR/αPD‐1‐MN Enhances Anticancer Efficacy and Suppresses Liver Metastasis

To directly compare the therapeutic efficacy of the FFX/SUR/αPD‐1‐MN group with the FFX/αPD‐1‐MN and SUR/αPD‐1‐MN groups, an orthotopic tumor model was established. The MN patches were applied directly onto the orthotopic tumors. High‐resolution images illustrating the MN morphology prior to application and during insertion into orthotopic tumors are provided in Figure S2A–F. Histological analysis of tumor sections revealed that the MN patches gradually degraded over time within the tumor tissue, and by 6 weeks post‐administration, the MN tips were almost completely degraded (Figure S3A–D). Consistently, the results demonstrated that the FFX/SUR/αPD‐1‐MN treatment achieved the most pronounced antitumor effect among all groups (Figure S4A–C).

To investigate the anticancer efficacy of FFX/SUR/αPD‐1‐MN in PDAC, we used Panc02 cells to establish subcutaneous and orthotopic implantation models in C57BL/6 mice (Figure 4A,D). The subcutaneous model provided a reproducible and easily monitored platform for rapid assessment of tumor growth inhibition, whereas the orthotopic model more faithfully mimicked the pancreatic tumor microenvironment, including stromal and immune components. Dose‐optimization experiments showed that a half‐dose MN patch was sufficient to achieve therapeutic efficacy comparable to that of full‐dose systemic administration; therefore, the 1/2 × MN‐loaded dose was selected for subsequent experiments (Figure S5A–C).

**FIGURE 4:**
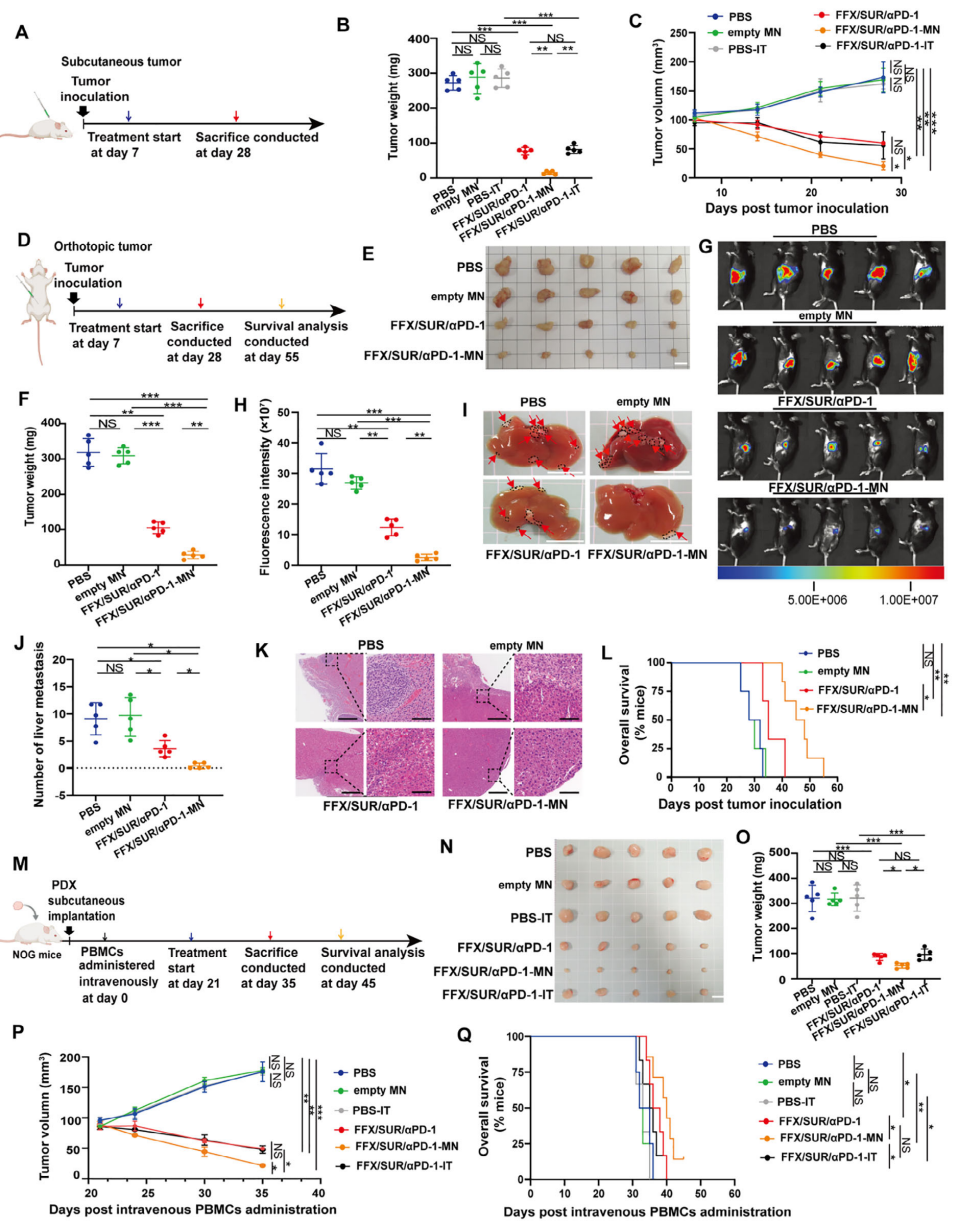
Therapeutic evaluation of FFX/SUR/αPD‐1‐MN patches in subcutaneous, orthotopic, and humanized PBMCs‐PDX PDAC models. (A) Panc02 mouse PDAC cells were subcutaneously injected into C57BL/6 mice. When tumors reached ∼100 mm^3^, five mice per group were treated with PBS, empty MN, intratumoral administration of PBS, FFX (i.p.) plus SUR (p.o.) plus αPD‐1 (i.p.) and empty MN, intratumoral administration of FFX plus SUR plus αPD‐1, or FFX/SUR/αPD‐1‐MN until the treatment endpoint. Schematic of the Panc02 subcutaneous model (Created with BioRender.com). (B) Tumor weights of Panc02 subcutaneous PDAC tumors from each group (*n* = 5 mice per group, biologically independent samples). (C) Growth curves of subcutaneous Panc02 tumors at the indicated time points (*n* = 5 mice per group, biologically independent samples). Tumor growth over time was analyzed using two‐way ANOVA, and statistical comparisons correspond to the final time point shown in panel C. (D) Schematic illustration of the anticancer experiment and survival monitoring in orthotopic Panc02‐luc tumor‐bearing mice (Created with BioRender.com). (E–H) Tumor images (scale bar, 10 mm) (E), tumor weights (F), in vivo fluorescence imaging (G), and fluorescence intensity (H) of orthotopic Panc02 tumor‐bearing mice at day 21 after treatment, as described in panel D (*n* = 5 mice per group, biologically independent samples). (I–K) Representative images of tumors in the liver (scale bars, 10 mm) (I), the number of tumor lesions (*n* = 5 mice per group, biologically independent samples) (J), and H&E staining of livers (Red arrows indicate tumor lesions (scale bars, 500 µm [left], 100 µm [right]) (K). (L) Kaplan–Meier survival plot of orthotopic Panc02 tumor‐bearing mice after the treatment indicated in panel D (*n* = 10 mice per group, biologically independent samples). (M) Schematic of the anticancer experiment and survival monitoring schedule for the humanized PBMCs‐PDX mouse model (Created with BioRender.com). (N, O) Representative tumor images (scale bar, 10 mm) (N), and tumor weights (O) from each treatment group (*n* = 5 mice per group, biologically independent samples). (P) Growth curves of tumors in humanized PBMCs‐PDX tumor‐bearing mice at the indicated time points (*n* = 5 mice per group, biologically independent samples). Tumor growth over time was analyzed using two‐way ANOVA, and statistical comparisons correspond to the final time point shown in panel P. (Q) Kaplan–Meier survival plot of humanized PBMCs‐PDX tumor‐bearing mice following the treatments described in panel M (*n* = 10 mice per group, biologically independent samples). Statistical analysis was performed using one‐way ANOVA (B, F, H, J, and O), two‐way ANOVA (C and P), and log‐rank test (L and Q). Data are presented as the mean ± SD. ^***^ *p* < 0.001, ^**^ *p* < 0.01, ^*^ *p* < 0.05; NS, not significant.

In the subcutaneous model, mice were divided into six groups and treated with PBS control; empty MN patches applied weekly; intratumoral administration of PBS; FFX (oxaliplatin 2 mg kg^−1^, irinotecan 20 mg kg^−1^, 5‐fluorouracil 20 mg kg^−1^, leucovorin 24 mg kg^−1^, weekly i.p.) plus SUR (20 mg kg^−1^, daily p.o.) plus αPD‐1 (10 mg kg^−1^, every three days i.p.) and empty MN patches; FFX/SUR/αPD‐1‐MN patches delivering 50% FFX, SUR and αPD‐1 drug equivalents weekly, with each mouse receiving two MN patches per application; and intratumoral administration of FFX (LOHP 1 mg kg^−1^, CPT‐11 10 mg kg^−1^, 5‐FU 10 mg kg^−1^, LV 12 mg kg^−1^, once weekly) combined with αPD‐1 (5 mg kg^−1^, every three days) and SUR (10 mg kg^−1^, daily), until the study endpoint. More significant inhibition of tumor growth was observed in the FFX/SUR/αPD‐1‐MN group at a half dose compared with the conventional systemic administration group, the intratumoral administration group, and other control groups in the subcutaneous model (Figure 4B,C and Figure S6A).

In the orthotopic PDAC model, intratumoral injection was not feasible because SUR requires once‐daily dosing, and daily laparotomy for pancreatic delivery is not permitted under animal welfare regulations. The MN platform overcomes this ethical barrier by eliminating the need for repeated daily laparotomy and enabling sustained intratumoral release from a single application. Consistent with the subcutaneous findings, the FFX/SUR/αPD‐1‐MN therapy produced the most pronounced antitumor effects (Figure 4E–H and Figure S6B). In addition, as shown in Figure 4I–L, a significant retardation of liver metastases, and prolonged survival rates were observed in the FFX/SUR/αPD‐1‐MN group compared with other groups. Moreover, compared with the control group, the FFX/SUR/αPD‐1‐MN therapy induced no obvious weight loss or hepatic and renal toxicity, whereas the conventional administration group exhibited the opposite effects (Figure S7A–E). Furthermore, a 28‐day treatment‐associated systemic toxicity study showed that FFX/SUR/αPD‐1‐MN treatment induced no detectable histopathological abnormalities in major organs and did not cause hepatic or renal toxicity, supporting its favorable safety profile and translational potential (Figure S8A–F). These findings suggest that compared with traditional systemic administration, FFX/SUR/αPD‐1‐MN marked antitumor efficacy, suppression of liver metastases, and low toxicity at half dose on PDAC models. This indicates that the MN system achieves a more effective pathway through the simultaneous delivery of drugs at multiple sites.

To further assess the therapeutic efficacy of FFX/SUR/αPD‐1‐MN in human PDAC, we established a humanized PDAC PDX model by implanting patient‐derived pancreatic tumor fragments into NOG mice, followed by intravenous administration of human PBMCs (HLA‐A^*^11:01). In this humanized system, application of the FFX/SUR/αPD‐1‐MN patch markedly inhibited tumor growth and significantly prolonged survival compared with control groups (Figure 4M–Q), demonstrating the therapeutic benefit of localized, sustained delivery in a human tumor background.

### 2.4 Pharmacokinetic Profiling of FFX/SUR/αPD‐1‐MN Patch in Vivo

Pharmacokinetic evaluations in orthotopic Panc02‐bearing C57BL/6 mice revealed distinct exposure profiles between the MN patch and systemic delivery groups. After administration, the concentration levels of 5‐fluorouracil (5‐FU), leucovorin (LV), irinotecan (CPT‐11), and oxaliplatin (LOHP), SUR, and αPD‐1 were detected in plasma at 5, 15, 30 min, and 1, 3, 10, 24, 72, 168 h. The systemic administration group exhibited rapid absorption with high peak plasma concentrations, followed by rapid clearance (Figure 5A). In contrast, MN patch delivery significantly attenuated peak concentrations and AUC_0‐168_ values (Figure 5B,C). These data collectively validate the MN platform's ability to reduce systemic peak exposures and mitigate toxicity risks associated with conventional administration routes.

**FIGURE 5:**
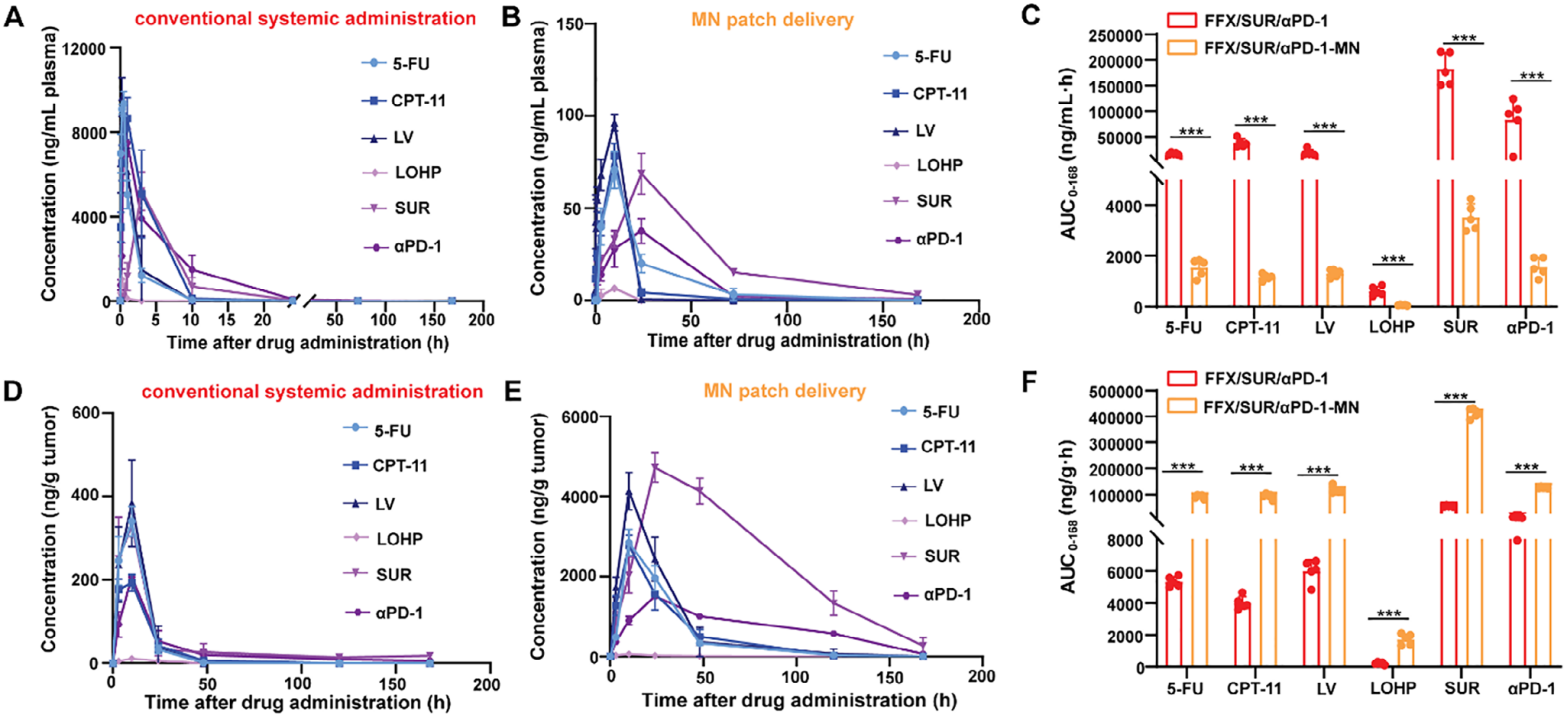
In vivo pharmacokinetic profiles of FFX/SUR/αPD‐1‐MN in the PDAC mouse model. (A, B) Plasma concentration‐time curves of FFX, SUR and αPD‐1 after systemic administration (A) vs. MN patch delivery (B) (*n* = 5 mice per group, biologically independent samples). (C) The area under the curve (AUC) in the range of 0–168 h in A and B (*n* = 5 mice per group, biologically independent samples). (D, E) Comparative pharmacokinetic profiles of FFX, SUR, and αPD‐1 in tumor tissue following systemic administration (D) vs. MN patch delivery (E) (*n* = 5 mice per group, biologically independent samples). (F) The AUC in the range of 0–168 h in D and E (*n* = 5 mice per group, biologically independent samples). Statistical analysis was performed using unpaired two‐tailed Student's *t*‐tests (C and F). Data are presented as the mean ± SD. ^***^ *p* < 0.001, ^**^ *p* < 0.01, ^*^ *p* < 0.05; NS, not significant.

Pharmacokinetic analysis in orthotopic Panc02 tumors demonstrated superior tumor‐targeted delivery via FFX/SUR/αPD‐1‐MN administration compared to systemic treatment. Systemic administration exhibited transient drug exposure with rapid peaks, followed by rapid clearance (Figure 5D). In contrast, MN delivery achieved sustained release kinetics, significantly enhancing tumor retention across all agents (Figure 5E,F). These data confirm MN‐driven pharmacokinetic optimization, enabling prolonged therapeutic thresholds while minimizing off‐target toxicity.

### 2.5 MN Delivery Enhances CD8^+^ T Cells and Inhibits Immunosuppressive Cells

Flow cytometric analysis of orthotopic Panc02 tumors in C57BL/6 mice was conducted to evaluate immune cell population dynamics following distinct administration regimens. The results demonstrated that CD8^+^ T cells exhibited a pronounced increase, while Foxp3^+^ regulatory T cells and CD206^+^ tumor‐associated macrophages infiltration were significantly lower in FFX/SUR/αPD‐1‐MN group compared to other groups, suggesting a potential reduction in immunosuppressive cell populations and enhanced antitumor immunity. No significant immune cell alterations were detected in the empty MN or PBS control groups, confirming that the MN material and puncturing procedure minimally impacted the tumor immune microenvironment (Figure 6A–F and Figures S9–S12). The observed changes were thus primarily attributable to therapeutic agents delivered via MNs. Collectively, these findings suggest that FFX/SUR/αPD‐1‐MN treatment promotes a favorable shift in the effector‐to‐suppressor cell ratio, potentially amplifying antitumor immune responses.

**FIGURE 6:**
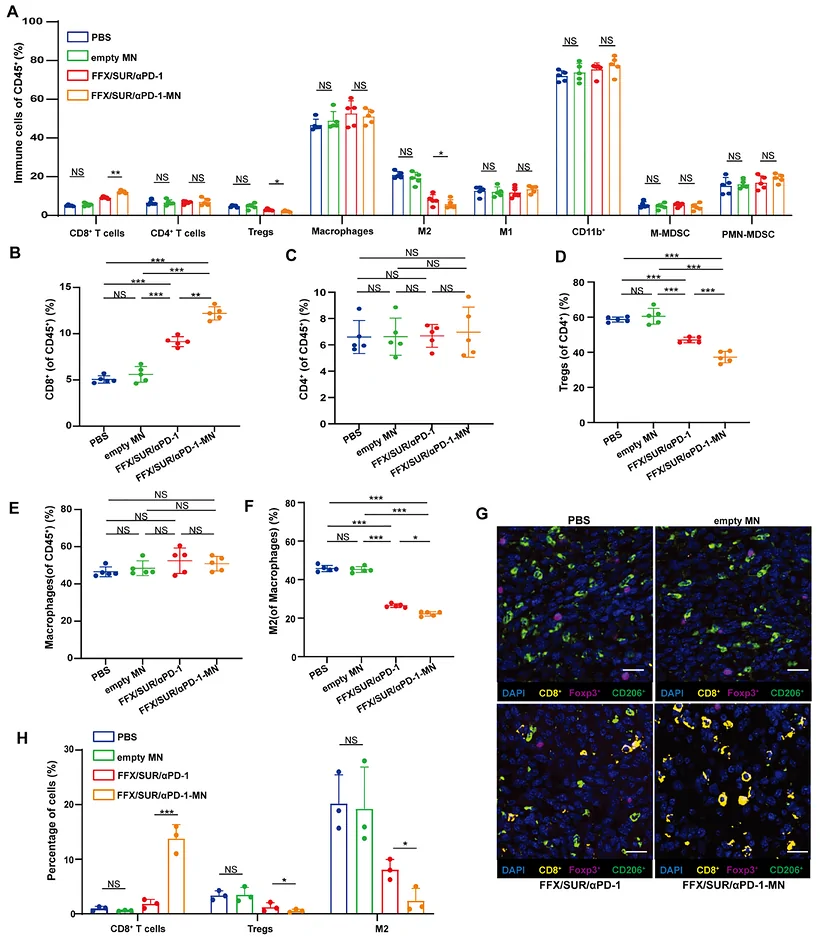
FFX/SUR/αPD‐1‐MN enhances CD8^+^ T cells and suppresses immunosuppressive cells infiltration in PDAC. (A–F) Flow cytometric analysis of immune cell profiles in the PDAC microenvironment at day 21 after treatment, as described in Figure 4D. Schematic representation of immune cell alterations across treatment groups (A) (*n* = 5 mice per group, biologically independent samples). The percentage of CD8^+^ T cells in CD45^+^ cells (B), CD4^+^ T cells in CD45^+^ cells (C), Tregs in CD4^+^ T cells (D), total macrophages in CD45^+^ cells (E), and M2 macrophages in total macrophages (F) (*n* = 5 mice per group, biologically independent samples). (G, H) Immunofluorescent staining visualized CD8^+^ T cells (yellow), Foxp3^+^ Tregs (rose red), and CD206^+^ M2 macrophages (green), with DAPI (blue) serving as a nuclear counterstain. This analysis revealed the spatial distribution and localization patterns of these cells within the tumor microenvironment (scale bars, 300 µm) (G), as well as the percentages of CD8^+^ T cells, Tregs, and M2 macrophages in total cells of every localization pattern (H) (*n* = 3, biologically independent samples). Statistical analysis was performed using unpaired two‐tailed Student's *t*‐tests (A and H) and one‐way ANOVA (B–F). Data are presented as the mean ± SD. ^***^ *p* < 0.001, ^**^ *p* < 0.01, ^*^ *p* < 0.05; NS, not significant.

Immunofluorescence staining further corroborated the flow cytometry findings. Tumors treated with FFX/SUR/αPD‐1‐MN demonstrated markedly enhanced CD8^+^ T cells infiltration, whereas Tregs and M2 macrophages exhibited significantly reduced infiltration (Figure 6G,H). This spatial reorganization suggests that FFX/SUR/αPD‐1‐MN therapy fosters a pro‐inflammatory tumor microenvironment conducive to effector T cell function while simultaneously attenuating immunosuppressive cell populations. Together, FFX/SUR/αPD‐1‐MN modulates both the spatial organization and relative abundance of key immune subsets in PDAC and contributes to the enhancement of antitumor immunity.

### 2.6 FFX/SUR/αPD‐1‐MN Reprograms the PDAC Tumor Microenvironment and Potentiates Therapeutic Efficacy

To further investigate the potential molecular mechanisms underlying MN‐mediated regulation of PDAC tumor growth and TME reprogramming, we performed RNA sequencing on orthotopic pancreatic tumors in the mouse model. Comparative analysis revealed a total of 798 differentially expressed genes (DEGs) in the FFX/SUR/αPD‐1‐MN group compared with the systemic treatment group (Figure 7A and Figure S13). Gene set variation analysis (GSVA) and heatmap results demonstrated that tumor cell death‐related pathways, and immune activation pathways exhibiting significant upregulation in the FFX/SUR/αPD‐1‐MN group (Figure 7B–F and Figures S14–S16). In contrast, apoptosis‐related markers demonstrated marked upregulation, while EMT‐related markers were significantly downregulated (Figure 7C,D). Furthermore, cytokines associated with immunosuppressive cell infiltration, particularly for Tregs and M2 macrophages, were found to be significantly downregulated in the FFX/SUR/αPD‐1‐MN treatment group (Figure 7E). Conversely, cytokines linked to tumoricidal immune cell infiltration (M1 macrophages and CD8^+^ T cells) demonstrated marked upregulation in the FFX/SUR/αPD‐1‐MN cohort (Figure 7F,G and Figure S16A). Western blot analysis further confirmed that the FFX/SUR/αPD‐1‐MN significantly suppressed tumor EMT and promoted tumor cell apoptosis more than other treatment groups (Figure 7H and Figure S17A). These results were further validated in human PDAC cells (Figure 7I and Figure S17B,C). Collectively, these findings suggest that MN‐mediated drug delivery can effectively inhibit tumor EMT, promote tumor cell apoptosis, and reverse the immunosuppressive TME characteristic of PDAC, thereby enhancing anti‐tumor efficacy.

**FIGURE 7:**
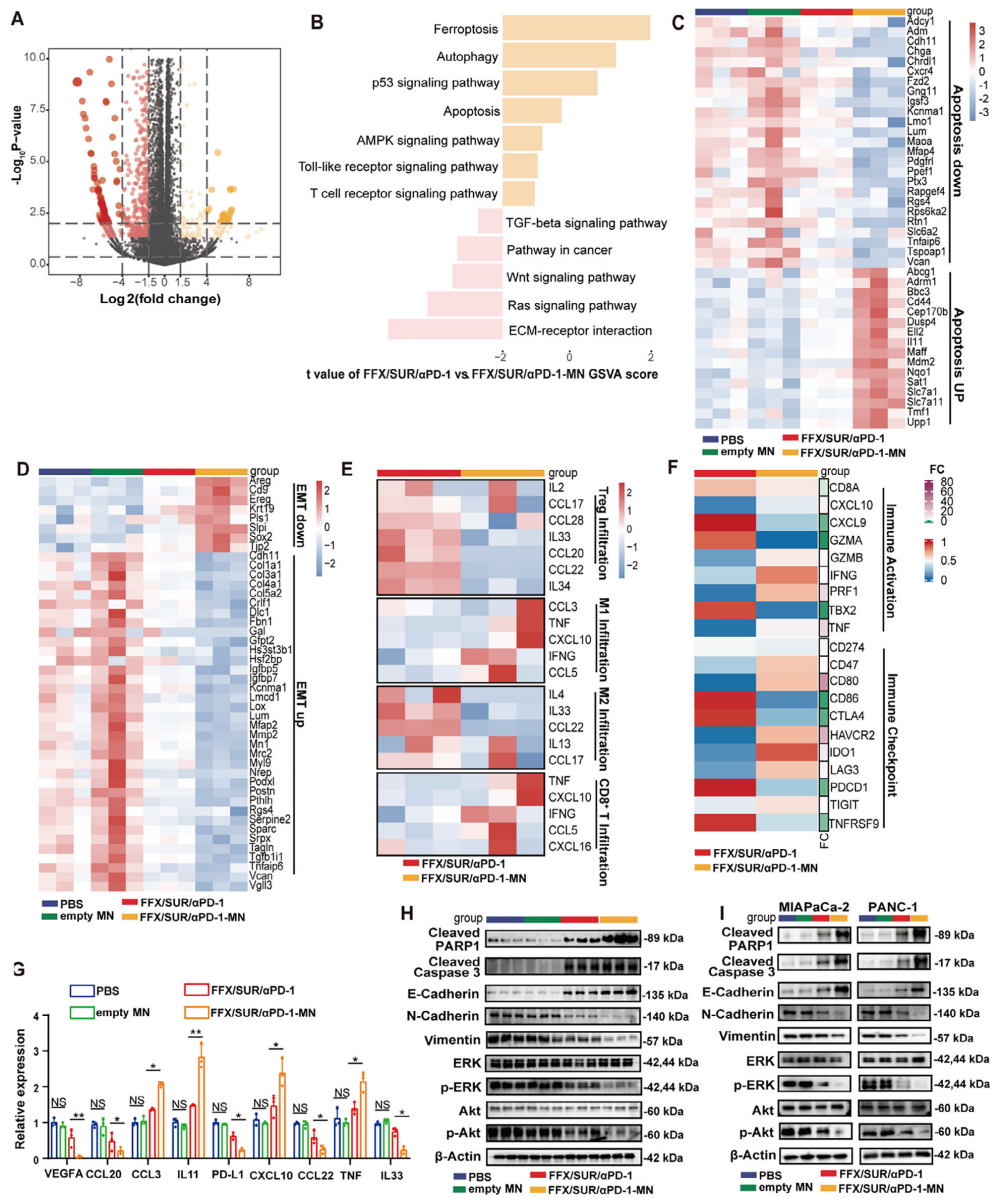
FFX/SUR/αPD‐1‐MN remodels the PDAC tumor microenvironment and amplifies antitumor efficacy. (A, B) The volcano plot (A) and pathway enrichment analysis (B) showing differences between FFX/SUR/αPD‐1‐MN and conventional systemic administration groups in the PDAC microenvironment at day 21 after treatment as described in Figure 4D (*n* = 3, biologically independent samples). (C, D) Heatmap displaying the gene expression analysis of apoptosis‐related pathways (C) and EMT‐related pathways (D) in different treatment groups (*n* = 3, biologically independent samples). (E, F) Cytokines associated with immunosuppressive cell infiltration (E), immune activation, and checkpoint (F) modulation in FFX/SUR/αPD‐1‐MN and systemic administration groups (*n* = 3, biologically independent samples). (G) mRNA expression of cytokines in PDAC tumors from different treatment groups (*n* = 3, biologically independent samples). (H, I) Western blot analysis of protein expression in tumor tissues (H) (*n* = 3, biologically independent samples) and human PDAC cells (I) from different treatment groups. Statistical analysis was performed using an unpaired two‐tailed Student's *t*‐test (G). Data are presented as the mean ± SD. ^***^ *p* < 0.001, ^**^ *p* < 0.01, ^*^ *p* < 0.05; NS, not significant.

## 3 Discussion

PDAC is anticipated to rank as the third most lethal malignancy [1], while conventional fluorouracil‐ or gemcitabine‐based induction therapies, with or without radiation, remain the first‐line treatment for unresectable cases. However, their limited clinical efficacy, suboptimal rates of conversion to surgical resection, and significant toxicity profiles collectively underscore the critical need for innovative induction strategies [2]. We developed an MN patch system integrating FFX, SUR, and αPD‐1 to overcome limitations in PDAC induction therapy via localized precision delivery and multi‐mechanism synergy. The FFX/SUR/αPD‐1 therapy demonstrated significantly enhanced antitumor efficacy compared with the dual‐combination regimens. The therapeutic platform was constructed as a bilayer core–shell MN system featuring distinct release kinetics. The outer layer was composed of a rapidly degradable matrix that enabled burst release of FFX within 24 h, regardless of whether the molecules were hydrophilic or lipophilic. The inner PVA‐based sustained‐release layer utilizes a cryogenic crosslinking mechanism, where multiple microcrystalline regions formed during freeze‐thaw cycles serve as physical crosslinking points, thereby creating a three‐dimensional network. Increased freeze‐thaw cycles enhance microcrystalline density, reduce matrix porosity, and decelerate drug release kinetics. The design enables sustained delivery of SUR and αPD‐1 over 7 days, thereby modulating the tumor microenvironment. The slower release is attributed to the lipophilicity of SUR and the large molecular weight of αPD‐1, combined with diffusion barriers imposed by the dense PVA network. Importantly, positioning FFX in the rapidly dissolving outer layer and embedding SUR and αPD‐1 in the inner sustained‐release core establishes a spatiotemporally coordinated therapeutic cascade. The initial burst release of FFX facilitates rapid tumor debulking, enhances antigen exposure, and transiently loosens stromal barriers, thereby improving the penetration and intratumoral distribution of subsequently released immunomodulatory agents. The sustained release of SUR and αPD‐1 further enables prolonged anti‐angiogenic and immune‐activating effects, which are essential for remodeling the immunosuppressive PDAC microenvironment and promoting effective T‐cell infiltration. Notably, this sequential strategy, in which FFX precedes SUR and αPD‐1, also aligns with current clinical practice, where cytotoxic chemotherapy is typically administered prior to anti‐angiogenic therapy and immune checkpoint blockade to potentiate therapeutic responsiveness. The MN‐mediated intratumoral delivery demonstrates superior pharmacokinetics compared to systemic administration, achieving over 8‐fold higher tumor area under the curve (AUC_0‐168_) and over 90% reduction in plasma peak concentration. This effectively lowers the systemic toxicity of chemotherapy drugs and confines the anti‐angiogenic effects of SUR to the tumor periphery, avoiding vascular integrity damage and related complications, such as hypertension and bleeding, caused by systemic VEGFR inhibition. The capability of enhancing localized efficacy through multisite MN delivery in the tumor and attenuating systemic toxicity enables low‐dose FFX/SUR/αPD‐1‐MN to achieve tumor regression effects equivalent to those of high‐dose systemic administration. Validation in a humanized PBMCs‐PDX mouse model further demonstrated that FFX/SUR/αPD‐1‐MN therapy can penetrate the dense human PDAC stroma and achieve robust antitumor activity and survival benefit.

Systemic chemotherapy for PDAC is often limited by severe toxicities, such as hepatotoxicity and diarrhea reported in the NASCA trial [13], which result from high systemic exposure. The MN platform presented here directly mitigates these limitations by delivering FFX, SUR, and αPD‐1 locally at significantly lower doses while maintaining potent antitumor activity. This approach reduces systemic drug levels and liver toxicity and enables multi‐point, spatially uniform intratumoral deposition through the array architecture‐an advantage not achievable with single‐site intratumoral injections. The programmed release profile, with rapid FFX release followed by sustained delivery of SUR and αPD‐1, further supports prolonged intratumoral exposure without dose escalation. Practically, MN administration does not require repeated laparotomy or laparoscopic ultrasound‐guided procedures, making it more clinically feasible than conventional intratumoral injection for PDAC. These combined features underscore the translational potential of FFX/SUR/αPD‐1‐MN‐based local therapy to improve tolerability and enhance treatment practicality in PDAC.

Immune checkpoint blockade (ICB) therapy, a significant breakthrough in cancer treatment, activates the adaptive immune system to mediate anti‐tumor responses and has shown remarkable efficacy in metastatic melanoma, renal cell carcinoma, and other solid tumors [32, 33, 34, 35]. Notably, although PD‐1 antibodies, alone or in combination with cytotoxic T‐lymphocyte–associated protein 4 (CTLA‐4) inhibitors, have become the standard treatment for advanced melanoma, their clinical efficacy in PDAC remains limited. Existing data show that the ORR of single‐agent ICB and anti‐PD‐1/anti‐CTLA‐4 combination therapy are only 0% and 3% [15, 16, 17], respectively, significantly lower than in other solid tumors. The poor responsiveness of PDAC to ICB is closely related to its unique TME, characterized by dense fibrotic stroma forming a physical barrier to drug penetration and an immunosuppressive microenvironment. In recent years, scientists have proposed various strategies to convert immunologically cold tumors into hot tumors, including tumor microenvironment modulation, T cell activation potentiation, combined immunotherapy regimens, and clinical trial design optimization. For example, targeting cancer‐associated fibroblasts (CAFs) and tumor‐associated macrophages (TAMs) to reprogram the TME and promote T cell infiltration using CD40 agonists and immune checkpoint inhibitors in combination to enhance T cell activity, and combining chimeric antigen receptor T (CAR‐T) cells therapy, vaccine therapy, and ICB to enhance anti‐tumor immune responses [36, 37, 38, 39, 40, 41, 42, 43, 44]. Although some strategies targeting stroma and myeloid cell inhibition have shown potential in improving T cell infiltration and prolonging survival in clinical trials, their overall efficacy still requires further optimization. Our MN delivery system overcomes the PDAC stromal barrier through physical penetration, enabling local high‐concentration PD‐1 antibody delivery and effectively blocking the interaction between tumor cell programmed death‐ligand 1 (PD‐L1) and T cell PD‐1. Additionally, postoperative recurrence of PDAC often stems from pre‐existing occult micrometastases. Traditional chemotherapy's efficiency in killing micrometastases is limited by uneven drug distribution and the immunosuppressive microenvironment. The FFX/SUR/αPD‐1‐MN regimen achieves synergistic control of the primary and micrometastatic lesions. These activated T cells can migrate through the circulatory system to common metastatic sites such as the liver, inhibiting the progression of micrometastases. Furthermore, the limited efficacy of adjuvant therapy after PDAC surgery is partly due to the rapid reconstruction of residual tumor cells in the immunosuppressive TME. This regimen reshapes the TME during the induction phase, increasing T cell infiltration and alleviating immunosuppression via reducing Tregs and M2 macrophages infiltration. FFX‐induced DNA damage enhances tumor cell sensitivity to subsequent radiotherapy/targeted therapy, creating favorable conditions for postoperative adjuvant immunotherapy or targeted therapy, overcoming the recurrence dilemma in PDAC treatment. Our study proposes an innovative multimodal therapeutic framework for PDAC that synergistically achieves enhanced oncological efficacy with a mitigated toxicity burden.

In clinical practice, the MNs in this configuration can be delivered to PDAC tumor sites via laparoscopic ultrasound guidance or particle implantation techniques. Standard 12‐mm trocars can readily accommodate the ∼0.5 cm^2^ MN patch without deformation, and the MNs’ high fracture strength (∼1.18 N per needle) and maximum pressure tolerance (16.7 mPa) provide sufficient robustness for surgical manipulation. However, the optimal implantation methodology, including depth control, spatial distribution patterns, and real‐time dosimetric monitoring, requires further clinical validation, particularly considering the complex tumor microenvironment and retroperitoneal anatomical constraints. Furthermore, the core–shell layer formulation and thickness of the MNs can be further customized to achieve on‐demand release patterns for different therapies. The materials used in the MNs are biocompatible and degradable, avoiding potential allergic reactions and toxicity issues [20, 45, 46, 47, 48, 49, 50, 51], and the patch does not require removal after application because the MNs naturally degrade in vivo. Benefiting from the physical crosslinking of PVA, the loading process avoids the use of organic solvents and elevated temperatures, preserving the bioactivity of the loaded drugs. This MN system can encapsulate various biological therapeutic agents requiring high doses and sensitivity to environmental factors, including hormones and vaccines.

## 4 Conclusions

In this study, we developed MN patches loaded with FFX, SUR, and αPD‐1, aiming to overcome the bottleneck in PDAC induction therapy through local precision delivery and a multi‐mechanism synergy strategy. The FFX/SUR/αPD‐1 therapy showed significantly higher anticancer efficacy compared to FFX plus αPD‐1 or SUR plus αPD‐1 combination. The MN technology enables time‐controlled drug release at multiple sites simultaneously around the tumor, reducing systemic toxicity while inducing immunogenic cell death, decreasing M2 macrophages and Tregs, and enhancing CD8^+^ T cells infiltration, thereby reversing the immunosuppressive microenvironment. The key advance of this work resides in the spatiotemporally controlled drug release, sustained intratumoral drug retention, and the strategic integration of FFX/SUR/αPD‐1 oncological mechanisms through chemo‐targeted‐immunotherapy coordination. Our research offers a paradigm of highly efficient, and low‐toxicity comprehensive treatment for PDAC.

## 5 Experimental Section

### 5.1 Fabrication of Core–Shell MNs

To fabricate MN patches, two solutions were cast sequentially onto the mold. For the core layer, PVA (Sigma–Aldrich, 363146) was dissolved in deionized water (20 mg mL^−1^) to form the base solution. SUR (MedChemExpress, HY‐12297) and PD‐1 antibodies (either a mouse αPD‐1 (STARTER, S0B0594, clone S‐5001) or a human αPD‐1 (MedChemExpress, HY‐P9971) were dispersed into the PVA solution at predefined ratios to achieve per‐patch loadings of approximately 0.70 mg SUR and 0.10 mg αPD‐1. The drug‐loaded PVA suspension was cast into polydimethylsiloxane (PDMS) molds (10 × 10 array), followed by centrifugation (3000 rpm, 10 min) and three freeze‐thaw cycles (‐20°C for 8 h freezing, 4°C for 4 h thawing) to induce crystallographic crosslinking. For the shell layer, a blend of PVP (Sigma–Aldrich, 856568) and PVA was prepared by dissolving 10 mg mL^−1^ of each polymer in deionized water. 5‐FU (MedChemExpress, HY‐90006), LV (TargetMol, T24403), CPT‐11 (MedChemExpress, HY‐16562) and LOHP (MedChemExpress, HY‐17371) were dispersed into the PVP/PVA matrix at optimized ratios to obtain per‐patch loadings of approximately 0.10 mg 5‐FU, 0.12 mg LV, 0.10 mg CPT‐11 and 0.01 mg L‐OHP (total FFX ≈ 0.33 mg per patch). This secondary suspension was cast into complementary PDMS molds. Core–shell structural alignment was achieved using a precision jig, followed by a secondary freeze‐thaw cycle. Finally, the MN patches were obtained through vacuum drying (32°C, 24 h) and gentle demolding. Control empty MNs were fabricated using identical procedures in the absence of therapeutic agents. For fluorescent layer differentiation, Nile red (Sigma–Aldrich, 19123) was incorporated into the core PVA solution, and fluorescein isothiocyanate (FITC) (Sigma–Aldrich, 46950) was incorporated into the shell PVP/PVA solution.

### 5.2 Characterizations of the FFX/SUR/αPD‐1‐MN Patch

The mechanical properties of the MN patches were evaluated using a displacement force testing apparatus (Force Gauge, Mark‐10, Copiague, NY). Specifically, an individual MN patch was mounted onto a vertical stainless‐steel platform with the MNs facing upward. The sensor probe of the test station was then advanced vertically toward the MNs at a rate of 0.1 mm s^−1^. Initially, the distance between the sensor and the MN tips was set at 1.0 cm. Force and displacement data were recorded starting from the moment the sensor made contact with the MN tips and continued until the sensor had moved 0.4 mm closer to the patch backing.

To assess uniformity, five MN patches from independent batches were individually dissolved in PBS (pH 7.4). The loading efficiency of the PD‐1 antibody was quantified using an enzyme‐linked immunosorbent assay (ELISA) with a Mouse αPD‐1 Detection Kit (Beyotime, PP778). For bioactivity validation, the functional activity of αPD‐1 in the MNs was compared with that of freshly prepared native αPD‐1. Stability studies were conducted under controlled desiccation conditions (25 ± 2°C, relative humidity < 15%) over a period of 24 weeks, with residual αPD‐1 levels assessed monthly using ELISA.

### 5.3 Cell Line and Cell Culture

Human PANC‐1 (SCSP‐535, CSTR:19375.09.3101HUMSCSP535), MIAPaCa‐2 (SCSP‐568, CSTR:19375.09.3101HUMSCSP568) and mouse Panc02 (SCSP‐5468, CSTR:19375.09.3101MOUSCSP5468) cells were purchased from the National Collection of Authenticated Cell Cultures. Cells were cultured in DMEM (Gibco, Thermo Scientific, Waltham, USA) supplemented with 10% fetal bovine serum (FBS, Gibco), penicillin (100 U mL^−1^), and streptomycin (100 µg mL^−1^), and cultured at 37°C in a humidified atmosphere containing 5% CO_2_. All cell lines used in this study were genotyped by short tandem repeat profiling, passaged fewer than 20 times for experiments, and routinely tested for mycoplasma contamination. The cell lines were confirmed contamination free.

### 5.4 In Vitro Release Studies of FFX/SUR/αPD‐1‐MN

The in vitro release of FFX, SUR, and αPD‐1 from MN patches was evaluated by immersing each patch in PBS as the release medium. The glass vessels containing the release media were incubated in a shaker water bath (37°C, 80 rpm). At predetermined time points (0, 2, 8, 24, 48, 72, 96, 120, and 168 h), aliquots of the release medium (1.0 mL) were collected and replaced with an equal volume of fresh medium to maintain sink conditions.

Quantification of FFX and SUR concentrations in the collected samples was performed using high‐performance liquid chromatography‐mass spectrometry (HPLC‐MS) with a Waters system (Milford, MA). Separation of CPT‐11 and SUR were achieved on a ZORBAX Eclipse Plus C_18_ column (50 mm × 2.1 mm; 3.5 µm particle size) maintained at 40°C. The mobile phase consisted of solvent A (water with 0.1% formic acid and 5 mm ammonium acetate) and solvent B (Acetonitrile). The gradient profile was programmed as follows: from 0.00 to 0.30 min, the composition was maintained at 80% solvent A and 20% solvent B; from 0.30 to 0.90 min, a linear gradient was applied, decreasing solvent A from 80% to 10% and increasing solvent B from 20% to 90%; from 0.90 to 1.90 min, the composition was held constant at 10% solvent A and 90% solvent B to ensure adequate separation of analytes; from 1.90 to 2.00 min, a rapid return to the initial conditions was achieved by increasing solvent A from 10% back to 80% and decreasing solvent B from 90% to 20%; and from 2.00 to 2.50 min, the system was equilibrated at the initial mobile phase composition (80% A, 20% B) to stabilize the column before the next injection. Samples (0.5 µL) were injected at a flow rate of 0.75 mL min^−1^, and mass spectrometric detection was performed using electrospray ionization (ESI) in positive ion mode. MS (ESI) *m/z*: [M + H]^+^ calcd for C_33_H_38_N_4_O_6_ (CPT‐11), 587.68; found, 587.530; [M + H]^+^ calcd for C_24_H_28_N_6_O_3_S (SUR), 481.58; found, 481.108.

Separation of 5‐FU, LV and LOHP were achieved on an Sepax Bio C_18_ column (150 mm × 4.6 mm; 3 µm particle size) maintained at 40°C. The mobile phase consisted of solvent A (water with 0.05% acetic acid and 2.5 mm ammonium acetate) and solvent B (Methanol). The gradient elution program was as follows: from 0.00 to 0.20 min, solvent A was maintained at 95% and solvent B at 5%; from 0.20 to 1.50 min, solvent A was linearly decreased to 15% while solvent B was linearly increased to 85%; from 1.50 to 4.70 min, solvent A was held constant at 15% and solvent B at 85% to ensure adequate separation of analytes; from 4.70 to 4.80 min, solvent A was rapidly restored to 95% and solvent B to 5%; finally, from 4.80 to 5.50 min, the system was maintained at the initial mobile phase composition (95% A, 5% B) to stabilize the column prior to the next injection. Samples (1 µL) were injected at a flow rate of 1.00 mL min^−1^, and mass spectrometric detection was performed using ESI in negative ion mode. MS (ESI) *m/z*: [M − H]^−^ calcd for C_4_H_3_FN_2_O_2_ (5‐FU), 129.08; found, 128.954; [M − H]^−^ calcd for C_20_H_23_N_7_O_7_ (LV), 472.44; found, 472.199; [M − H]^−^ calcd for C_8_H_14_N_2_O_4_Pt (LOHP), 396.29; found, 396.092.

### 5.5 Cytotoxicity Assay

Cytotoxicity assay of MN patches was performed using Panc02 cells. Cells were seeded into 96‐well plates at a density of 1 × 10^4^ cells per well and cultured in 100 µL of DMEM (Gibco, Thermo Scientific, Waltham, USA) with 10% fetal bovine growth serum (FBS; Gibco). After incubation at 37°C in 5% CO_2_ for 24 h, PBS, empty MN patch, or FFX/SUR/αPD‐1‐MN patch were added to the respective wells. After incubation with MN patch for an additional 24 h, the medium in each well was removed, and the wells were gently washed with PBS, followed by incubation with 90 µL of fresh DMEM and 10 µL of CCK‐8 solution (lot no. TH531) for 2 h. The absorbance was measured at 450 nm using a Synergy H1Microplate Reader (Bio Tek).

### 5.6 Animal Studies

All C57BL/6 mice (4–6‐week‐old male) were obtained from Shanghai Slac Laboratory Animal Co., and NOG mice (5–6‐week‐old female) were obtained from Charles River Laboratories and fed in a pathogen‐free vivarium under standard conditions. Surgical procedures were conducted under the guidelines approved by the Ethics Boards of Shanghai General Hospital (Shanghai, China). The animal (2023AW029) and human (2022‐109) experimental protocols were approved by the Ethics Boards of Shanghai General Hospital.

To establish the subcutaneous PDAC model, tumors were induced by subcutaneous injection of Panc02 cells (5 × 10^6^ cells in 100 µL PBS) into the right flank. When tumors reached ∼100 mm^3^ (calculated as a × b^2^/2, where a = longitudinal diameter and b = transverse diameter) [52, 53], mice were randomized into six groups: (1) PBS control group; (2) empty MN control group received weekly at tumor sites; (3) intratumoral administration of PBS; (4) conventional systemic administration group received weekly intraperitoneal FFX (LOHP 2 mg kg^−1^, CPT‐11 20 mg kg^−1^, 5‐FU 20 mg kg^−1^, LV 24 mg kg^−1^), every three days intraperitoneal PD‐1 antibody (10 mg kg^−1^), daily oral SUR (20 mg kg^−1^), and weekly empty MN patches; (5) FFX/SUR/αPD‐1‐MN group received weekly at tumor sites with each mouse administered two MN patches per application; (6) intratumoral administration group received FFX (LOHP 1 mg kg^−1^, CPT‐11 10 mg kg^−1^, 5‐FU 10 mg kg^−1^, LV 12 mg kg^−1^) once weekly, PD‐1 antibody (5 mg kg^−1^) every three days, and SUR (10 mg kg^−1^) daily. Tumor volumes were measured weekly using digital calipers, and body weight was recorded to evaluate systemic toxicity.

For the orthotopic model, mice were anesthetized, and a small flank incision was made to expose the pancreas. Subsequently, Panc02 cells stably expressing Luciferase (Panc02‐luc) (5 × 10^6^ cells in 30 µL PBS) were injected into the pancreatic parenchyma, followed by suturing. One week after implantation of Panc02 cells, mice were divided into 4 groups and received the same treatments as the subcutaneous model cohorts, except for the intratumoral injection group. MN patches were applied to orthotopic pancreatic tumors under direct visualization during laparotomy. Gentle compression was maintained for 15–30 s to ensure complete insertion of the MN, which subsequently remained stably anchored within the tumor parenchyma. The thin PVA backing layer exhibited intrinsic hydrophilicity and developed mild tackiness upon brief contact with moisture, enabling stable adhesion to the tumor surface. After insertion, the backing layer was left in place, and no removal was required. Death was defined as natural death during the study period or euthanasia required upon meeting any of the following criteria: maximum tumor diameter > 1.5 cm, > 20% loss of body weight, development of ascites, severe lethargy or hunching, or a moribund condition.

For the PDX mouse model [54, 55, 56, 57, 58, 59, 60], human PDAC tissues were obtained from Shanghai General Hospital (Shanghai, China) with written informed consent from all patients. The use of human specimens in this study was approved by the institutional ethics committee (2022‐109). First, the cryopreserved PDX tumors were recovered and subcutaneously inoculated onto the bilateral flanks of NOG mice, and the resulting tumors were harvested after 2–3 weeks and dissected into fragments approximately 2 mm in diameter. These freshly collected tumor pieces were then promptly processed and embedded in antibiotic‐supplemented Matrigel Matrix (Corning) for subsequent implantation. The tumor fragments were subcutaneously implanted into 5–6‐week‐old NOG mice within 2 h of surgical removal. Peripheral blood mononuclear cells (PBMCs) were purchased from Sailybio (Catalog No. XW0120351W), and 5 × 10^6^ human PBMCs (HLA‐A^*^11:01) were administered to each mouse via tail‐vein injection. Following PBMCs infusion, mice were monitored weekly for human T‐cell reconstitution, assessed by the percentage of hCD45^+^ cells in peripheral blood. Peripheral blood was collected from the retro‐orbital venous plexus of mice into EDTA‐coated EP tubes. A total of 50 µL of blood was mixed with 1 mL of 1 × RBC lysis buffer (diluted to 1 × with ddH_2_O; BD Pharmingen, 555899) and incubated at room temperature for 10 min. Samples were centrifuged at 1500 rpm for 5 min, the supernatant was discarded, and the cell pellet was washed with FACS buffer (PBS containing 2% FBS). Cells were first incubated with Fixable Viability Stain 440UV (BD Pharmingen, 566332) for 10 min at room temperature and washed with FACS buffer. Surface staining was performed by incubating the cells with PE‐Cy7 anti‐human CD45 (BD Pharmingen, 557748) for 30 min at 4°C. After staining, cells were washed twice and resuspended in FACS buffer for acquisition. Flow cytometry was performed on a BD Fortessa instrument, and data were analyzed using FlowJo v10.8.1. Treatment was initiated once the proportion of hCD45^+^ cells reached ≥25% and the tumors had grown to ∼100 mm^3^.

### 5.7 Histological Evaluation of MN Patch Degradation in Tumor Tissue

To evaluate the in situ degradation of MN patches within tumors, tumor tissues were harvested at 0 h, 2 weeks, 4 weeks, and 6 weeks after MN patch administration. Collected tumors were fixed in 4% paraformaldehyde and then embedded in paraffin. Histological analyses were performed using hematoxylin and eosin (H&E) staining to visualize the MN structures and surrounding tissue. Sections were examined under the microscope (KR PharmTech, FRP3) to assess the gradual degradation of MN tips over time.

### 5.8 Pharmacokinetic Studies

Pharmacokinetic studies were performed in the orthotopic model two weeks after Panc02 implantation. Mice were divided into a systemic treatment group and an FFX/SUR/αPD‐1‐MN group. Blood samples (∼0.1 mL) were collected in heparin‐treated tube via the oculi chorioideae vein at the predetermined time point 0, 0.083, 0.25, 0.5, 1.0, 3.0, 10.0, 24.0, 72.0, 168.0 h. Plasma was separated by centrifuging (2000 × g, 15 min, 4°C). For plasma analysis, plasma (50 µL) was mixed with acetonitrile (100 µL), vortexed (1 min), and centrifuged (15 400 × g, 10 min, 4°C). The resulting supernatant was then combined with 0.2% (v/v) formic acid in water (50 µL) prior to injection for analysis. Tissue samples were collected at multiple time points post‐administration (3.0, 10.0, 24.0, 48.0, 120.0 and 168.0 h) by euthanizing the mouse using CO_2_ asphyxiation and excising the orthotopic pancreatic tumor tissues. Tissue samples were homogenized to obtain homogenates (100 µL), which were mixed with methanol (200 µL), vortexed (1 min), and centrifuged (15 400 × g, 10 min, 4°C). Subsequently, the supernatant (100 µL) was combined with 0.2% (v/v) formic acid in water (50 µL), vortexed, and injected for analysis. Drug concentrations in both plasma and tumor tissues were measured using HPLC‐MS as previously described. The experimental period lasted for 7 days.

Pharmacokinetic parameters were determined using noncompartmental analysis with Phoenix WinNonlin v.8 (Certara, Princeton, NJ). The calculated parameters included C_max_ (the observed maximum plasma concentration), T_max_ (the time at which C_max_ was achieved), AUC_0–t_ (the area under the plasma concentration‐time curve from time zero to the time of the last quantifiable concentration, Clast, calculated using the linear trapezoidal rule).

### 5.9 Flow Cytometry Analysis

Tumor tissues were enzymatically dissociated through mechanical mincing followed by digestion with collagenase IV (1 mg mL^−1^; Sigma, C5138) and DNase I (200 U mL^−1^; Sigma, D4527) in RPMI‐1640 medium at 37°C for 45 min with agitation. Cell suspensions were filtered through 100 µm strainers and subjected to Percoll gradient centrifugation (40%/80%, 2500 rpm, 20 min). Isolated leukocytes were washed in PBS/1% FBS staining buffer and adjusted to 1 × 10^7^ cells mL^−1^. Surface markers were labeled using fluorochrome‐conjugated antibodies against CD45 BV510 (1:100; BioLegend, 103137), CD3 BV421 (1:100; BD Biosciences, 562600), CD4 FITC (1:200; Thermo Fisher Scientific, 11‐0042‐85), CD8 PerCP‐Cy5.5 (1:100; BioLegend, 100734), CD11b BUV395 (1:100; Thermo Fisher Scientific, 363‐0112‐82), F4/80 APC (1:100; Thermo Fisher Scientific, 17‐4801‐82), Ly6G BV421 (1:100; BioLegend, 127627), Ly6C PerCP‐Cy5.5 (1:100; BioLegend, 128012), CD86 FITC (1:100; Thermo Fisher Scientific, 11‐0862‐85), Foxp3 PE (1:100; 12‐5773‐82), and CD206 PE (1:100; BioLegend, 141706). All samples were analyzed on a BD Fortessa X20 flow cytometer and analyzed using FlowJo (TreeStar).

### 5.10 Immunofluorescence

Tumor tissues were collected from Panc02‐derived orthotopic PDAC‐bearing mice immediately after sacrifice and fixed in 4% paraformaldehyde (PFA) at 4°C for 24 h. Fixed tissues were subsequently dehydrated through a graded ethanol series, cleared in xylene, and embedded in paraffin. Serial sections (4 µm thick) were cut using a rotary microtome and mounted on glass slides. Paraffin sections were subjected to deparaffinization through three sequential xylene immersions (5 min each), followed by stepwise rehydration in a graded ethanol series (100%, 95%, 80%, 70%) for 3 min per concentration, and subsequently rinsed with PBS. Antigen retrieval was carried out in citrate buffer (pH 6.0) at 95°C–100°C for 20 min, followed by cooling at room temperature for 20 min.

Following antigen retrieval, sections were incubated in blocking solution (5% bovine serum albumin in PBS) for 30 min at room temperature to prevent nonspecific binding. Primary antibodies against CD8A (1:200; Thermo Fisher Scientific, MA1‐10301), Foxp3 (1:200; Thermo Fisher Scientific, PA1‐16876), and CD206 (1:200; Thermo Fisher Scientific, PA5‐101657) were applied overnight at 4°C in a humidified chamber. Slides were then washed three times with PBS and incubated with the corresponding fluorescently labeled secondary antibodies (Alexa Fluor 570, 690, and 520; Thermo Fisher Scientific) for 1 h at room temperature in the dark. After washing in PBS, sections were counterstained with 4′,6‐diamidino‐2‐phenylindole (DAPI) for nuclear visualization. Coverslips were mounted using an antifade mounting medium (Vector Laboratories), and images were captured using a fluorescence or confocal microscope (Leica or Zeiss) under identical exposure settings. Image analysis and quantification were performed using ImageJ (NIH) or an equivalent software, ensuring consistent thresholding and region‐of‐interest definitions for accurate comparison across samples.

### 5.11 RNA Sequencing and Bioinformatic Analysis

Total RNA was extracted from tumor tissue collected from mice treated with PBS, empty MN, FFX (i.p.) plus SUR (p.o.) plus αPD‐1 (i.p.), and empty MN, or FFX/SUR/αPD‐1‐MN, using the RNeasy Mini Kit (Qiagen). RNA quality was assessed using an Agilent 2100 Bioanalyzer.

RNA sequencing (RNA‐seq) was performed on an Illumina HiSeq 4000 platform. Raw sequencing data were processed and aligned to the mouse genome (mm39) using STAR software. Gene expression was quantified using HTSeq. Differential expression gene (DEGs) analysis was performed using DESeq2, with genes meeting the criteria of absolute Log_2_FoldChange (|LogFC|) ≥ 1.5 and adjusted *p*‐value ≤ 0.05 considered statistically significant. Gene Set Variation Analysis (GSVA) was conducted using the GSVA R package (version 1.52.3) to investigate pathway‐level heterogeneity and biological behavior across different treatment groups. For this analysis, we utilized the ‘kegg.mmu.keg.genesets.gmt’ gene set collection downloaded from the Molecular Signatures Database (MSigDB), which was implemented within the GSVA package.

### 5.12 RT‐qPCR Assays

To quantify gene expression levels, cryopreserved specimens underwent RNA isolation employing Trizol reagent (Invitrogen, California, USA), followed by synthesis of cDNA through reverse transcription. Quantitative amplification was conducted using SYBR Green PCR Master Mix (DBI Bioscience, Ludwigshafen, Germany) on an ABI PRISM 7900 Sequence Detection System (Applied Biosystems), with β‐actin serving as the endogenous reference for data normalization.

Primer list:

mCCL20‐F: GCCTCTCGTACATACAGACGC

mCCL20‐R: CCAGTTCTGCTTTGGATCAGC

mVEGFA‐F: AGGGCAGAATCATCACGAAGT

mVEGFA‐R: AGGGTCTCGATTGGATGGCA

mTNF‐F: CAGGCGGTGCCTATGTCTC

mTNF‐R: CGATCACCCCGAAGTTCAGTAG

mCCL3‐F: CTCCCAGCCAGGTGTCATTTT

mCCL3‐R: CTTGGACCCAGGTCTCTTTGG

mCXCL10‐F: CCAAGTGCTGCCGTCATTTTC

mCXCL10‐R: GGCTCGCAGGGATGATTTCAA

mIL11‐F: GCGCTGTTCTCCTAACCCG

mIL11‐R: GAGTCCAGACTGTGATCTCCG

mPD‐L1‐F: GCTCCAAAGGACTTGTACGTG

mPD‐L1‐R: TGATCTGAAGGGCAGCATTTC

mCCL22‐F: CTCTGCCATCACGTTTAGTGAA

mCCL22‐R: GACGGTTATCAAAACAACGCC

mIL33‐F: ATTTCCCCGGCAAAGTTCAG

mIL33‐R: AACGGAGTCTCATGCAGTAGA

mGAPDH‐F: GCAGTGGCAAAGTGGAGATT

mGAPDH‐R: GAATTTGCCGTGAGTGGAGT

### 5.13 Western Blot Assay

Cellular lysates were prepared using RIPA lysis buffer supplemented with a protease inhibitor cocktail (Roche Diagnostics, Indianapolis, IN, USA). Protein samples underwent electrophoretic separation through discontinuous SDS‐PAGE gels before being electrophoretically transferred onto polyvinylidene difluoride (PVDF) membranes. Membranes were initially blocked with 5% non‐fat dried milk solution to prevent nonspecific binding. Subsequent incubations involved specific primary antibodies followed by species‐matched HRP‐conjugated secondary antibodies. Immunoreactive bands were visualized using enhanced chemiluminescence substrate, with digital image acquisition performed using the Image Quant LAS 4000 biomolecular imager (GE Healthcare Life Sciences, San Diego, CA, USA). The quantification of the Western blot was measured using Image J software.

The following primary antibodies from Proteintech Group were utilized for immunoblotting: Cleaved PARP1 (60555‐1‐Ig), Cleaved Caspase 3 (68773‐1‐Ig), E‐cadherin (20874‐1‐AP), N‐cadherin (22018‐1‐AP), and Vimentin (10366‐1‐AP); signaling pathway components ERK (11257‐1‐AP), phospho‐ERK (28733‐1‐AP), AKT (10176‐2‐AP), and phospho‐AKT (66444‐1‐Ig). Beta Actin (66009‐1‐Ig) served as the loading control. Corresponding secondary antibodies included HRP‐conjugated Goat Anti‐Rabbit IgG (SA00001‐2) and Goat Anti‐Mouse IgG (SA00001‐1), both sourced from Proteintech Group. Antibody dilutions were optimized according to manufacturer recommendations and preliminary validation experiments.

### 5.14 Statistics Analysis

Data are expressed as mean ± SD. The biological replicate or sample size (*n*) for each statistical analysis was shown in the figure legends. Intergroup comparisons were performed using Student's *t*‐test for two groups, one‐way ANOVA for multiple groups, and two‐way ANOVA for time‐course tumor growth experiments. Survival analysis was conducted using Kaplan–Meier estimators and compared by log‐rank testing. A *p*‐value < 0.05 was considered statistically significant for all tests. Statistical analyses were performed using GraphPad Prism version 10.2.0, and bioinformatics analyses were conducted using R version 4.4.1.

## Author Contributions

**Tingting Kong**: conceptualization, methodology, data curation, investigation, formal analysis, visualization, writing – original draft, writing – review and editing, supervision. **Ximo Xu**: investigation, formal analysis, visualization. **Xiao Zhang**: investigation, formal analysis. **Chuntao Wu**: investigation. **Zhengjun Qiu**: investigation. **Beiyuan Hu**: investigation, funding acquisition. **Zihao Qi**: investigation, funding acquisition. **Qiang Tian**: investigation, funding acquisition. **Yuqin Yang**: investigation, methodology. **Hanguang Dong**: investigation, methodology. **Fei Wu**: investigation, writing – review and editing. **Tuo Jin**: investigation, writing – review and editing. **Yan Zheng**: methodology, funding acquisition, writing – review and editing, supervision, conceptualization. **Jiang Long**: conceptualization, methodology, supervision, writing – review and editing, funding acquisition.

## Ethics Statement

The animal (2023AW029) and human (2022‐109) experimental protocols were approved by the Ethics Boards of Shanghai General Hospital.

## Consent

Human PDAC tissues were obtained from Shanghai General Hospital (Shanghai, China) with written informed consent from all patients.

## Conflicts of Interest

The authors declare no conflicts of interest.

## Supporting information




**Supporting File**: advs76478‐sup‐0001‐SuppMat.docx.

## Data Availability

The raw data of RNA‐sequencing has been deposited in the NCBI SRA database (PRJNA1279402). The data that support the findings of this study are available from the corresponding author upon reasonable request.
