# Supplementary material for: A Spatially Directed Microneedle Patch Enables Intratumoral Co‐Delivery of FOLFIRINOX, Surufatinib, and Anti‐PD‐1 for Chemo‐Immunotherapy of Pancreatic Ductal Adenocarcinoma
Source: Adv Sci (Weinh). 2026 Jul 13:e76478. Online ahead of print. doi: 10.1002/advs.76478 (PMC13360130; doi:10.1002/advs.76478)
Supplement: Supplementary file 1 — Supporting File: advs76478‐sup‐0001‐SuppMat.docx. [file ADVS-9999-e76478-s001.docx]

Supporting Information

A Spatially Directed Microneedle Patch Enables Intratumoral Co-Delivery of FOLFIRINOX, Surufatinib, and Anti-PD-1 for Chemo-Immunotherapy of Pancreatic Ductal Adenocarcinoma

*Tingting Kong, Ximo Xu, Xiao Zhang, Chuntao Wu, Zhengjun Qiu, Beiyuan Hu, Zihao Qi, Qiang Tian, Yuqin Yang***, Hanguang Dong***, Fei Wu***, Tuo Jin, Yan Zheng***, Jiang Long**


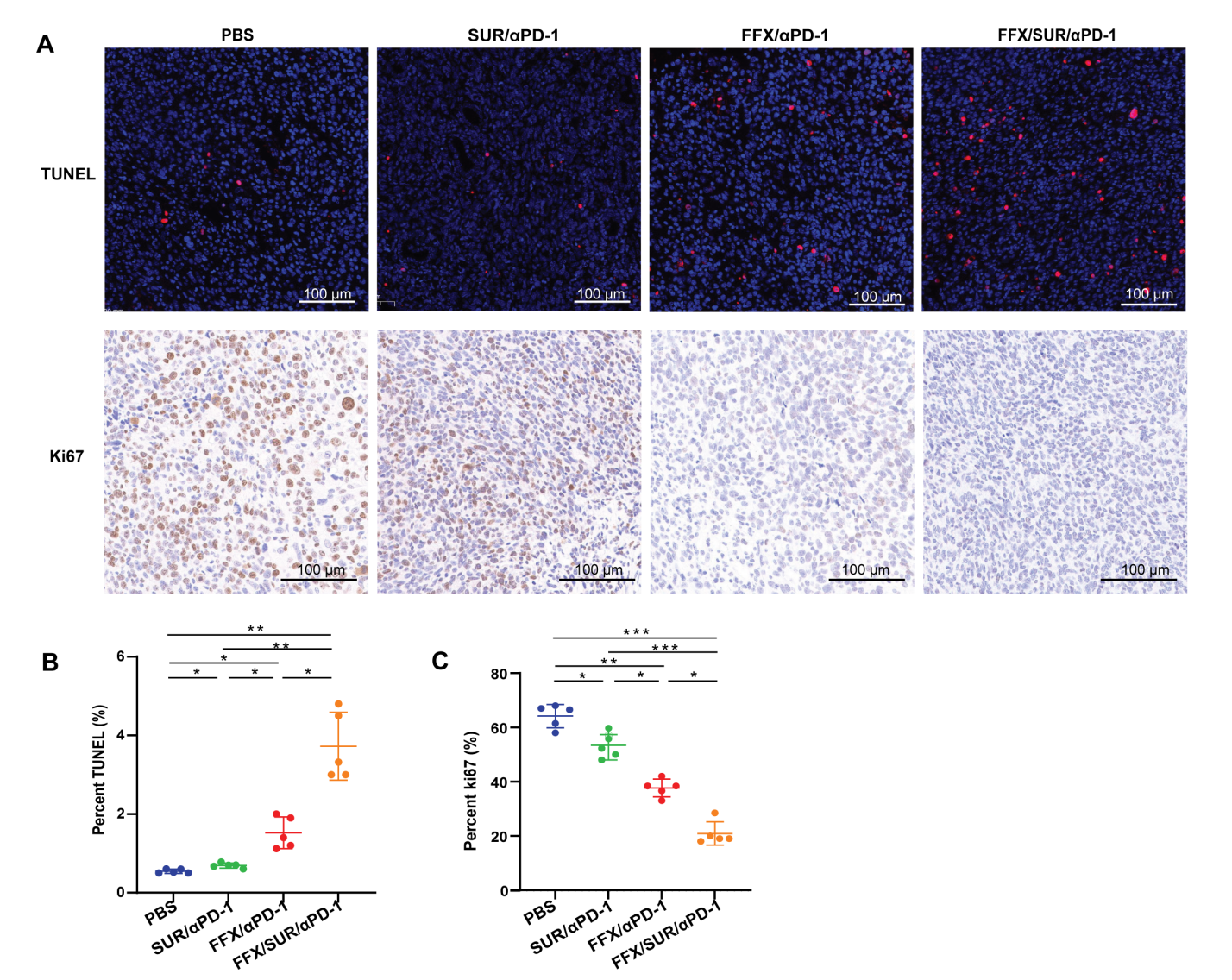


Figure S1. FFX/SUR/αPD-1 therapy enhances antitumor efficacy in orthotopic Panc02 tumors as evidenced by TUNEL and Ki67 staining. (A) TUNEL and Ki67 staining of tumor sections from PBS, SUR/αPD-1, FFX/αPD-1, and FFX/SUR/αPD-1 groups at day 28 after tumor inoculation as described in Figure 1A. (B-C) Quantification of TUNEL^+^ apoptotic cells (B) and Ki67^+^ proliferating cells in total cells of every localization pattern (C) (*n* = 5 mice per group, biologically independent samples). Statistical analysis was performed using one-way ANOVA (B and C). Data are presented as the mean ± SD. ****P* < 0.001, ***P* < 0.01, **P* < 0.05; NS, not significant.


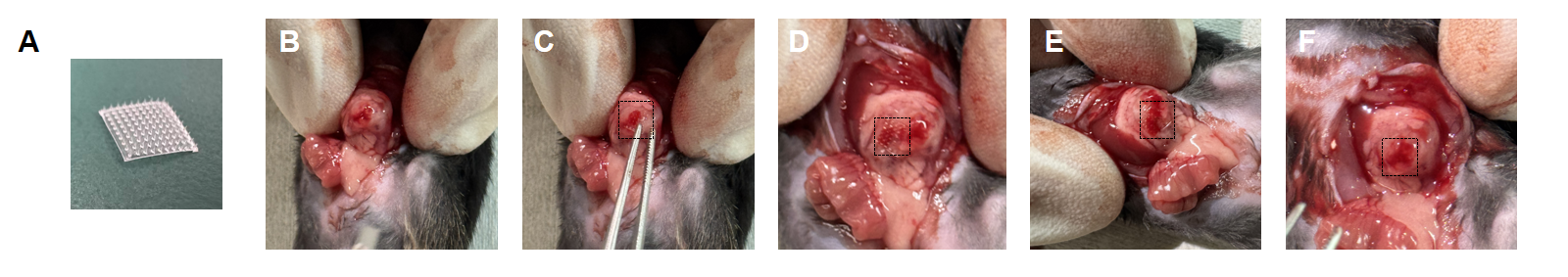


Figure S2. MN morphology before application (A) and during insertion into orthotopic Panc02 tumors (B-F).


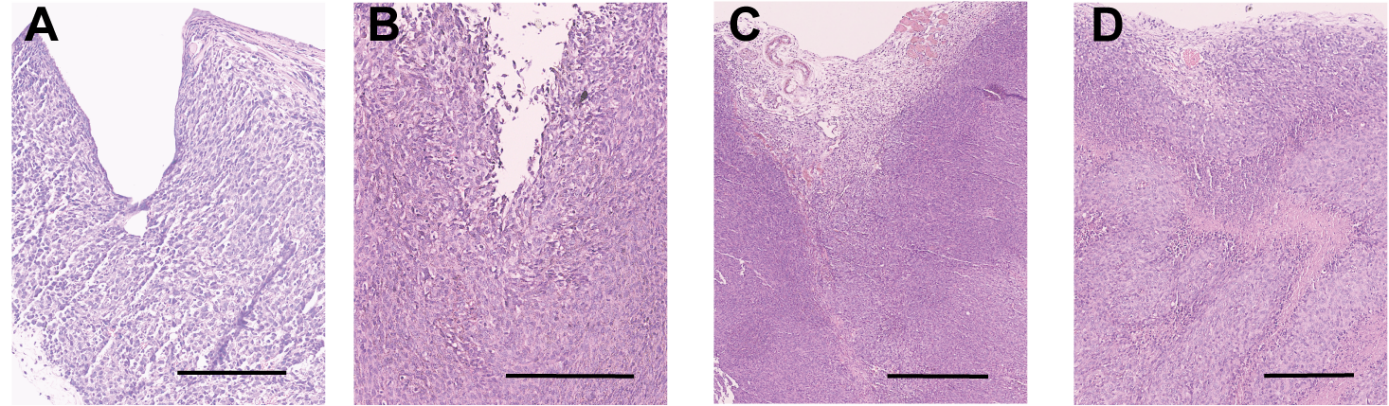


Figure S3. Evaluation of FFX/SUR/αPD-1-MN patch degradation in tumor tissue. (A-D) Tumor sections were collected at 0 hour (A), 2 weeks (B), 4 weeks (C), and 6 weeks (D) after MN patch administration and subjected to histological analysis (scale bars, 200 μm). Representative sections show the gradual degradation of MN tips over time.


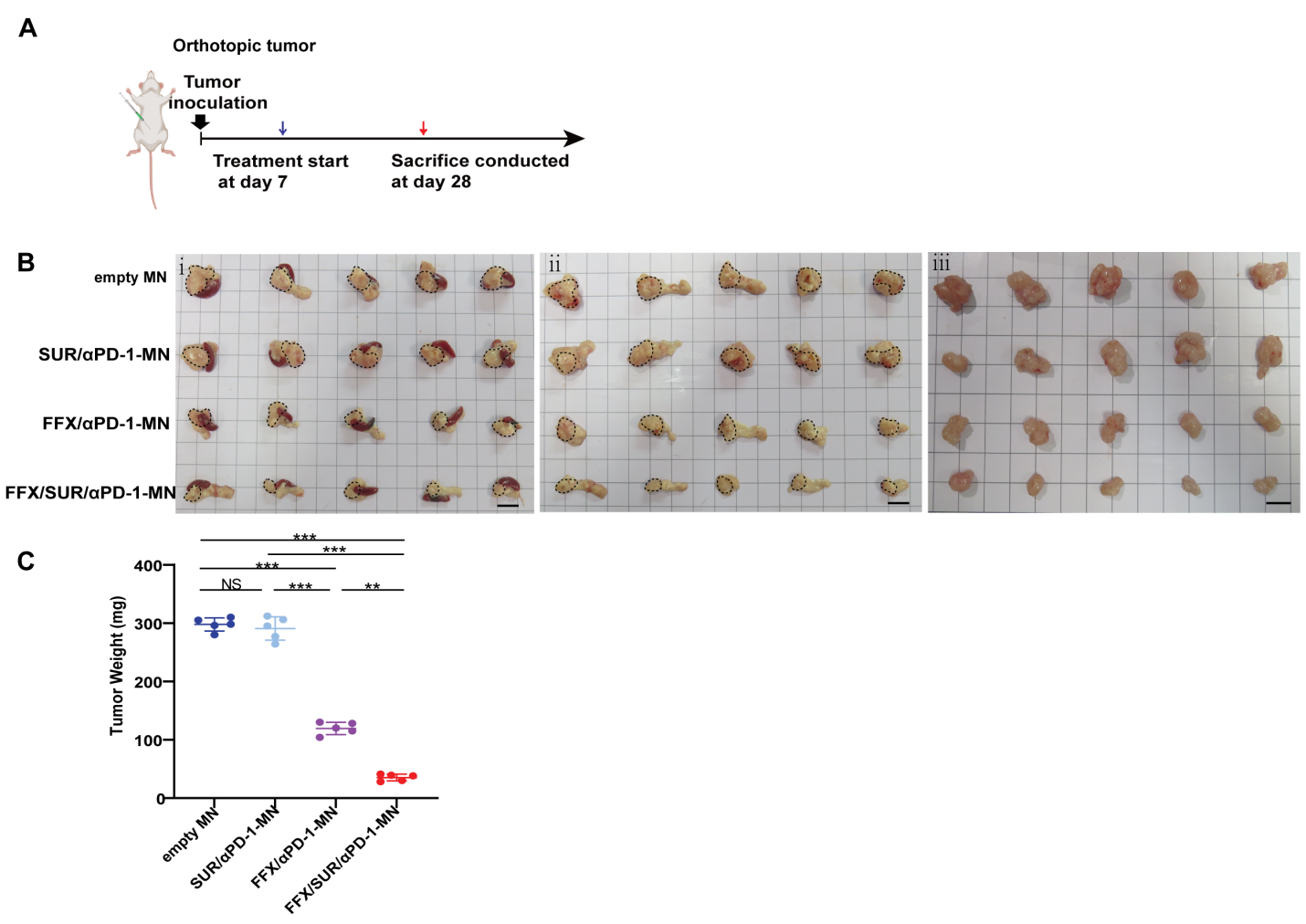


Figure S4. Anticancer evaluation of MN-based therapies in orthotopic Panc02 tumor-bearing mice. (A) Schematic illustration of the anticancer experiment in orthotopic Panc02 tumor-bearing mice (Created with BioRender.com). Five mice per group were treated with empty MN, SUR/αPD-1-MN, FFX/αPD-1-MN, or FFX/SUR/αPD-1-MN until the treatment endpoint. (B) Images of tumors collected at the endpoint from each treatment group, showing (i) pancreas with spleen attached, (ii) pancreas bearing the orthotopic tumor, and (iii) the excised tumor after dissection (scale bars, 10 mm). (C) Tumor weights from each treatment group (*n* = 5 mice per group, biologically independent samples). Statistical analysis was performed using one-way ANOVA (C). Data are presented as the mean ± SD. ****P* < 0.001, ***P* < 0.01, **P* < 0.05; NS, not significant.


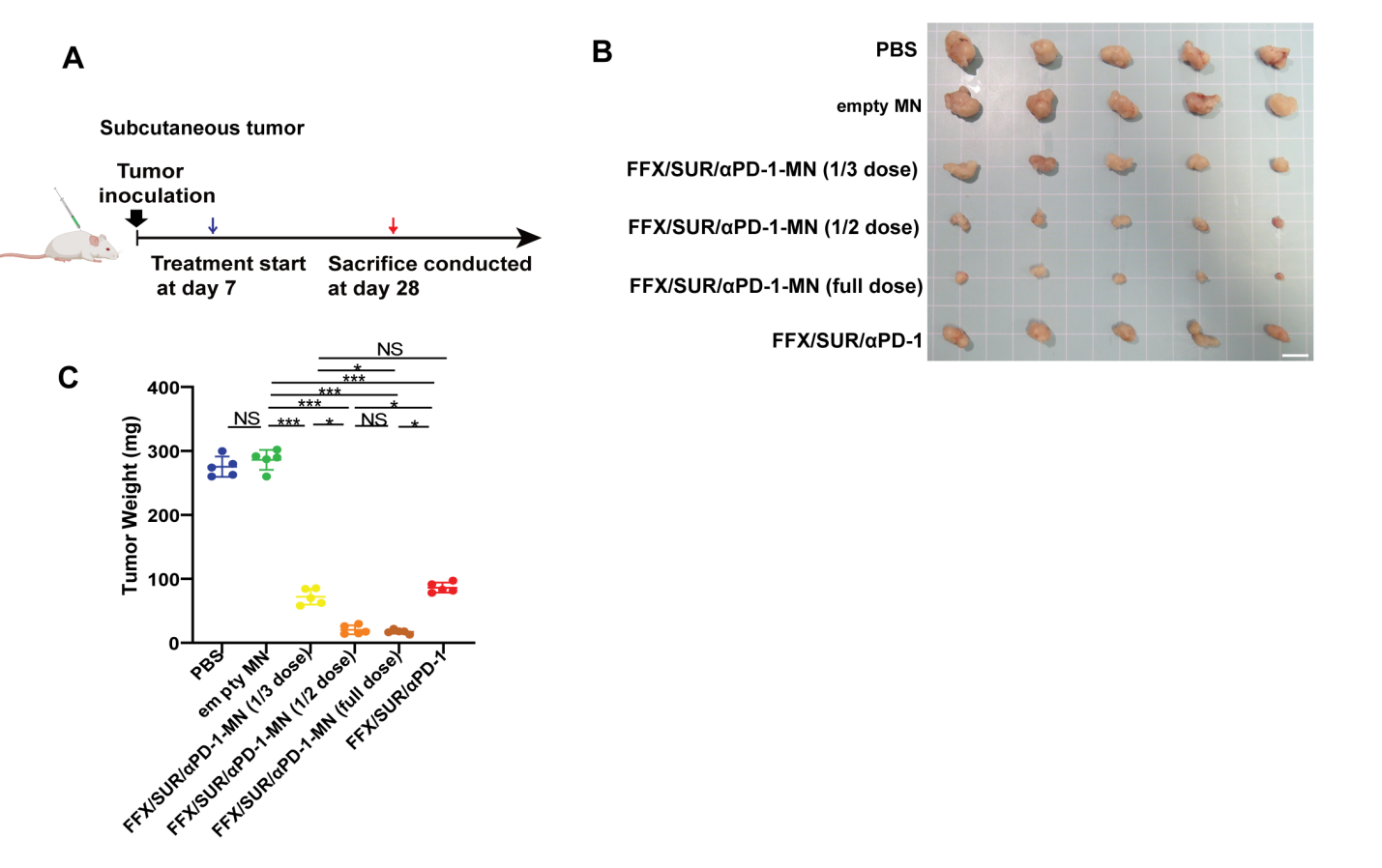


Figure S5. Dose-dependent evaluation of FFX/SUR/αPD-1-MN patches in the subcutaneous Panc02 model. (A) Panc02 mouse PDAC cells were subcutaneously injected into C57BL/6 mice. When tumors reached ~100 mm³, five mice per group were treated with PBS, empty MN, systemic FFX/SUR/αPD-1 with empty MN, or FFX/SUR/αPD-1-MN patches (full dose, half dose, or one-third dose equivalents of FFX/SUR/αPD-1) until the treatment endpoint. Schematic of treatment protocol for the subcutaneous model (Created with BioRender.com). (B-C) Tumor images (scale bar, 10 mm) (B) and tumor weights (C) from each treatment group (*n* = 5 mice per group, biologically independent samples). Statistical analysis was performed using one-way ANOVA (C). Data are presented as the mean ± SD. ****P* < 0.001, ***P* < 0.01, **P* < 0.05; NS, not significant.


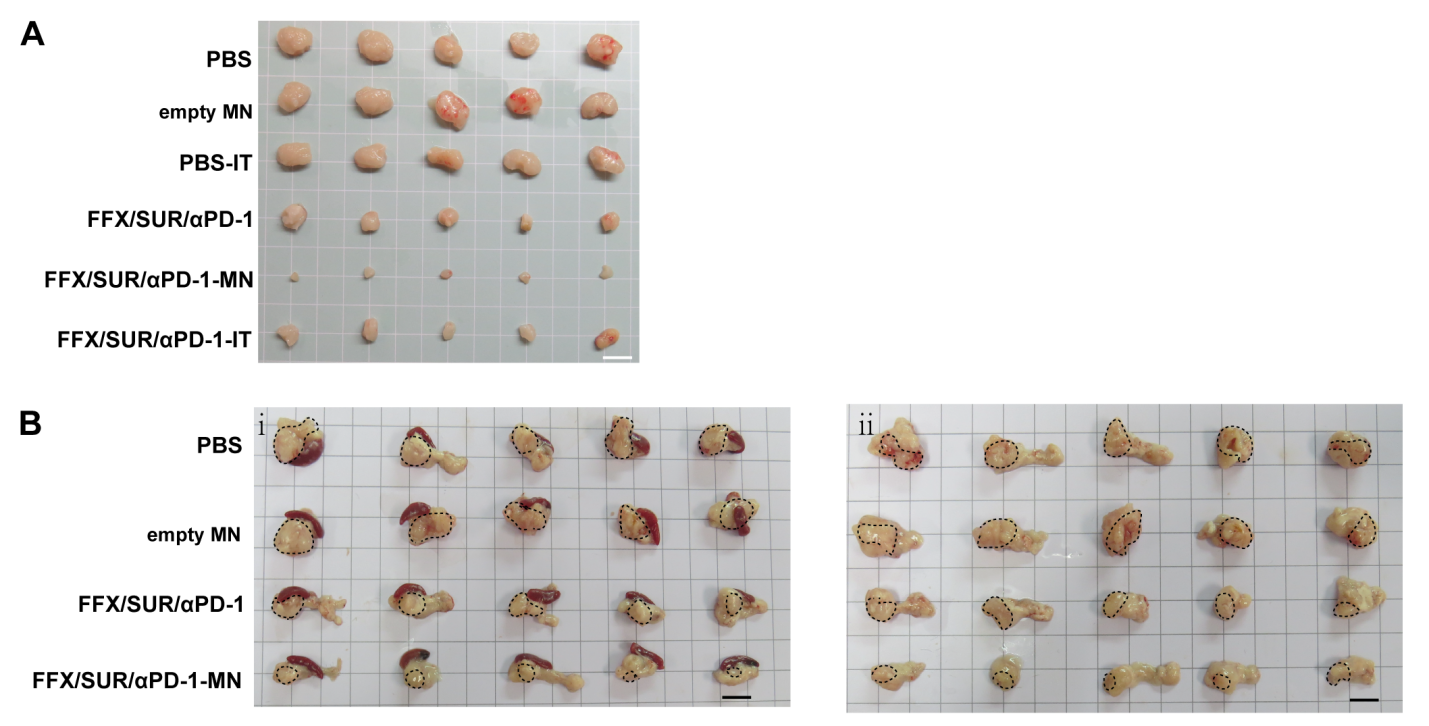


Figure S6. Therapeutic evaluation of FFX/SUR/αPD-1-MN patches in subcutaneous and orthotopic PDAC models. (A) Tumor images of the subcutaneous Panc02 tumor-bearing mice at 21 days after treatment as described in Figure 4A (scale bars, 10 mm; *n* = 5 mice per group, biologically independent samples). (B) Tumor images showing (i) the pancreas with the spleen attached and (ii) the pancreas bearing the orthotopic tumor at day 21 after treatment, as described in Figure 4D (scale bars, 10 mm; *n* = 5 mice per group, biologically independent samples).


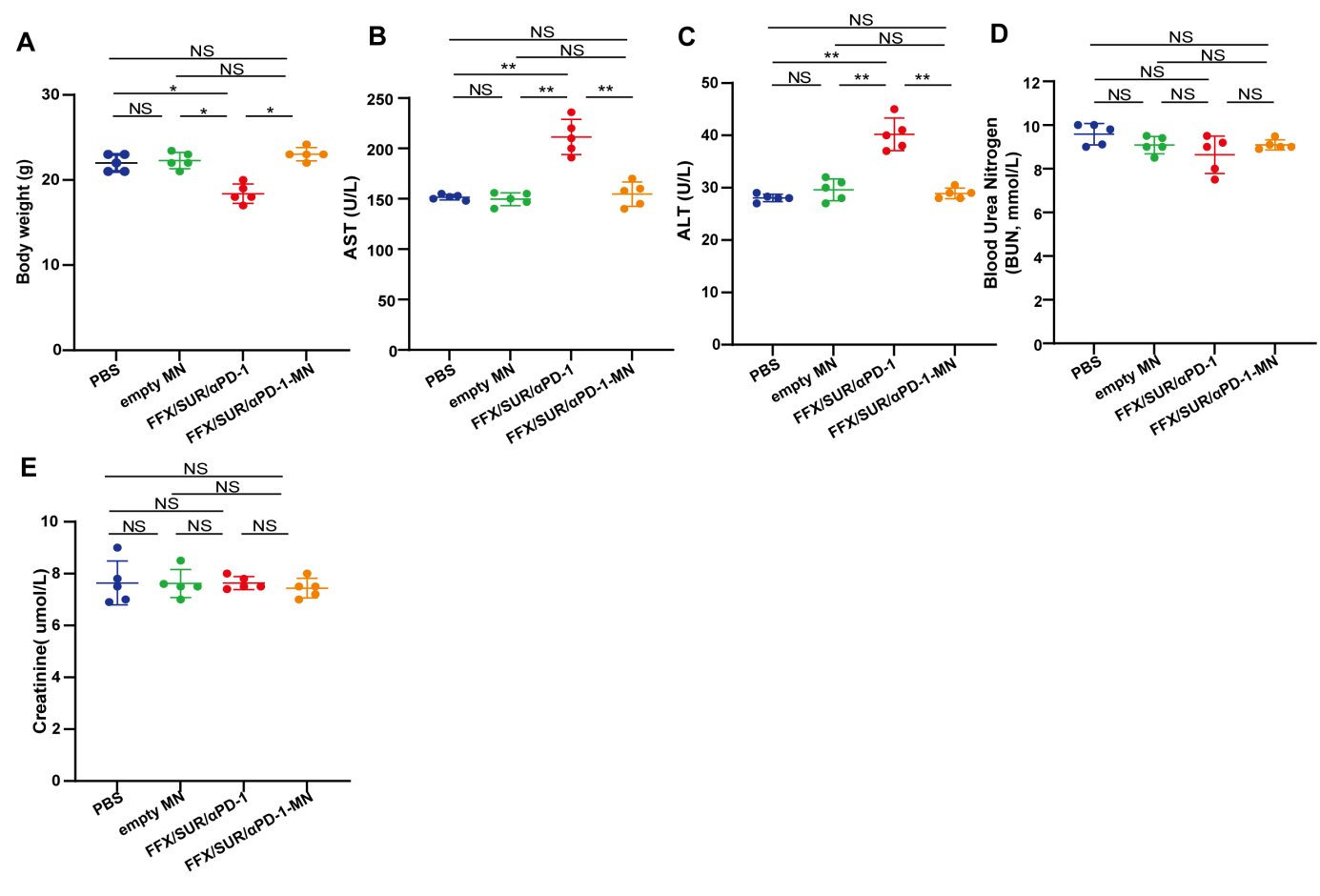


Figure S7. The safety profiles of FFX/SUR/αPD-1-MN therapy in orthotopic Panc02 tumor-bearing C57BL/6 mice. (A-E) The body weight (A), AST (B), ALT (C), blood urea nitrogen (D), and creatinine (E) levels of each group at day 21 after treatment as described in Figure 4D (*n* = 5 mice per group, biologically independent samples). Statistical analysis was performed using one-way ANOVA (A-E). Data are presented as the mean ± SD. ****P* < 0.001, ***P* < 0.01, **P* < 0.05; NS, not significant.


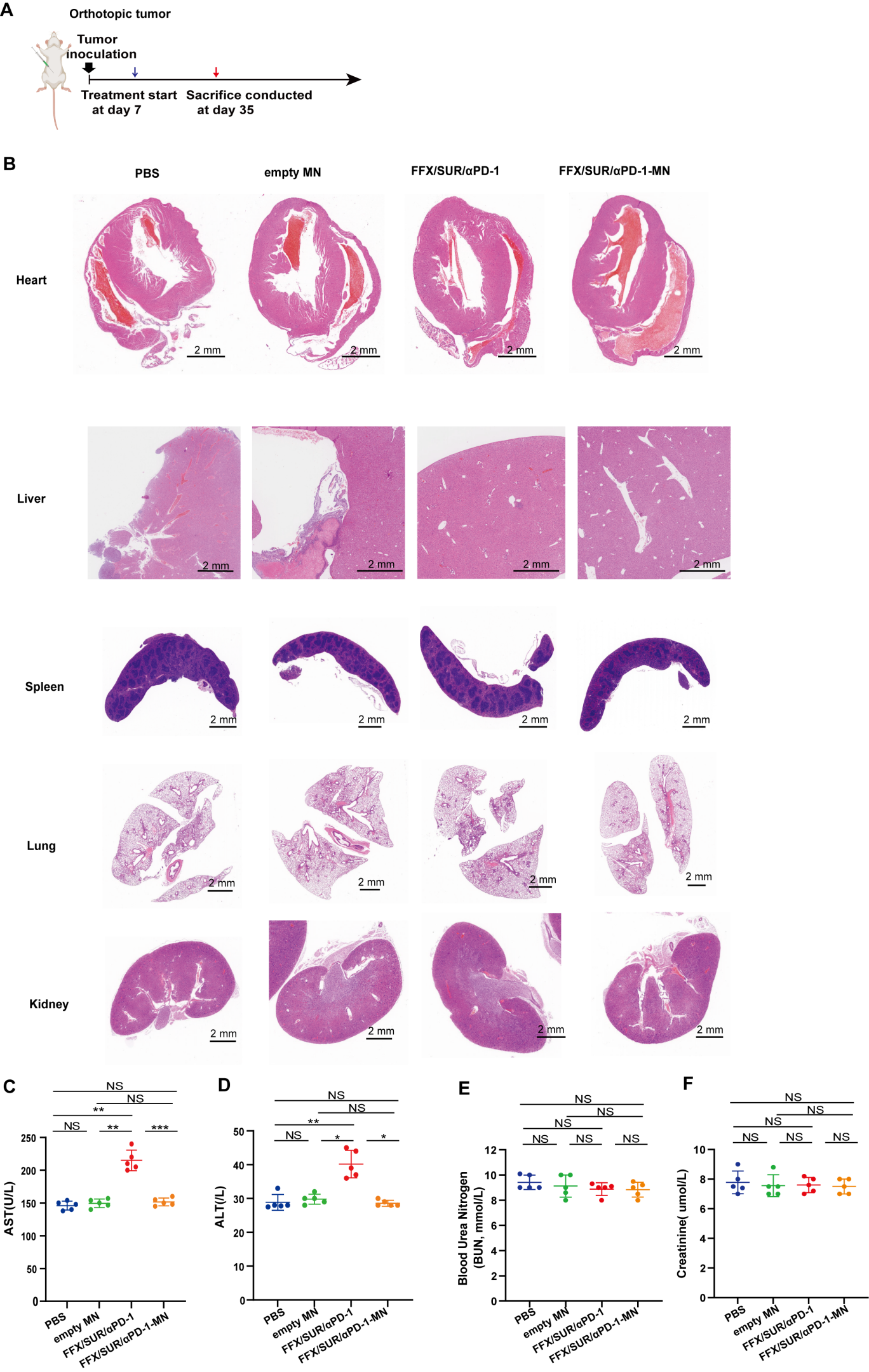


Figure S8. H&E staining of major organs and serum biochemical analysis in the 28-day treatment-associated systemic toxicity study. (A) C57BL/6 mice were orthotopically implanted with Panc02 cells. Schematic illustration of the treatment protocol for the orthotopic PDAC mouse model (Created with BioRender.com). Mice were treated with PBS, empty MN, systemic FFX (i.p.) plus SUR (p.o.) plus αPD-1 (i.p.) and empty MN, or FFX/SUR/αPD-1-MN until the treatment endpoint. (B) H&E staining of the major organs in orthotopic Panc02 tumor-bearing mice at day 28 after treatment, as indicated in panel A. (C–F) Serum levels of AST (C), ALT (D), blood urea nitrogen (E), and creatinine (F) in the same mice, measured at day 28 after treatment (*n* = 5 mice per group, biologically independent samples). Statistical analysis was performed using one-way ANOVA (C-F). Data are presented as the mean ± SD. ****P* < 0.001, ***P* < 0.01, **P* < 0.05; NS, not significant.


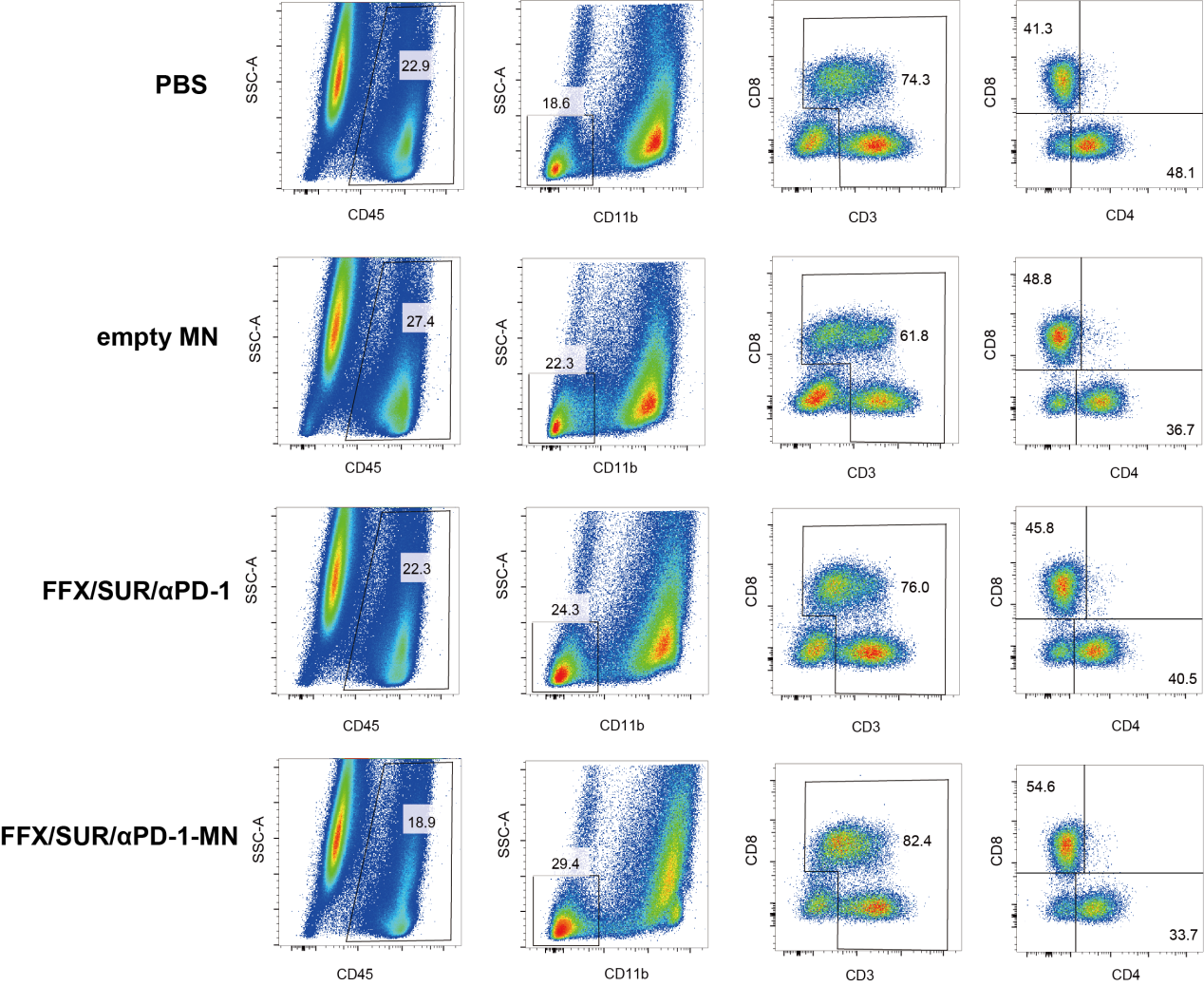


Figure S9. Flow cytometry gating strategy and representative plots showing the percentages of CD8^+^ and CD4^+^ T cells in CD45^+^ cells in the PDAC tumor microenvironment on day 21 after treatment, as shown in Figure 4D.


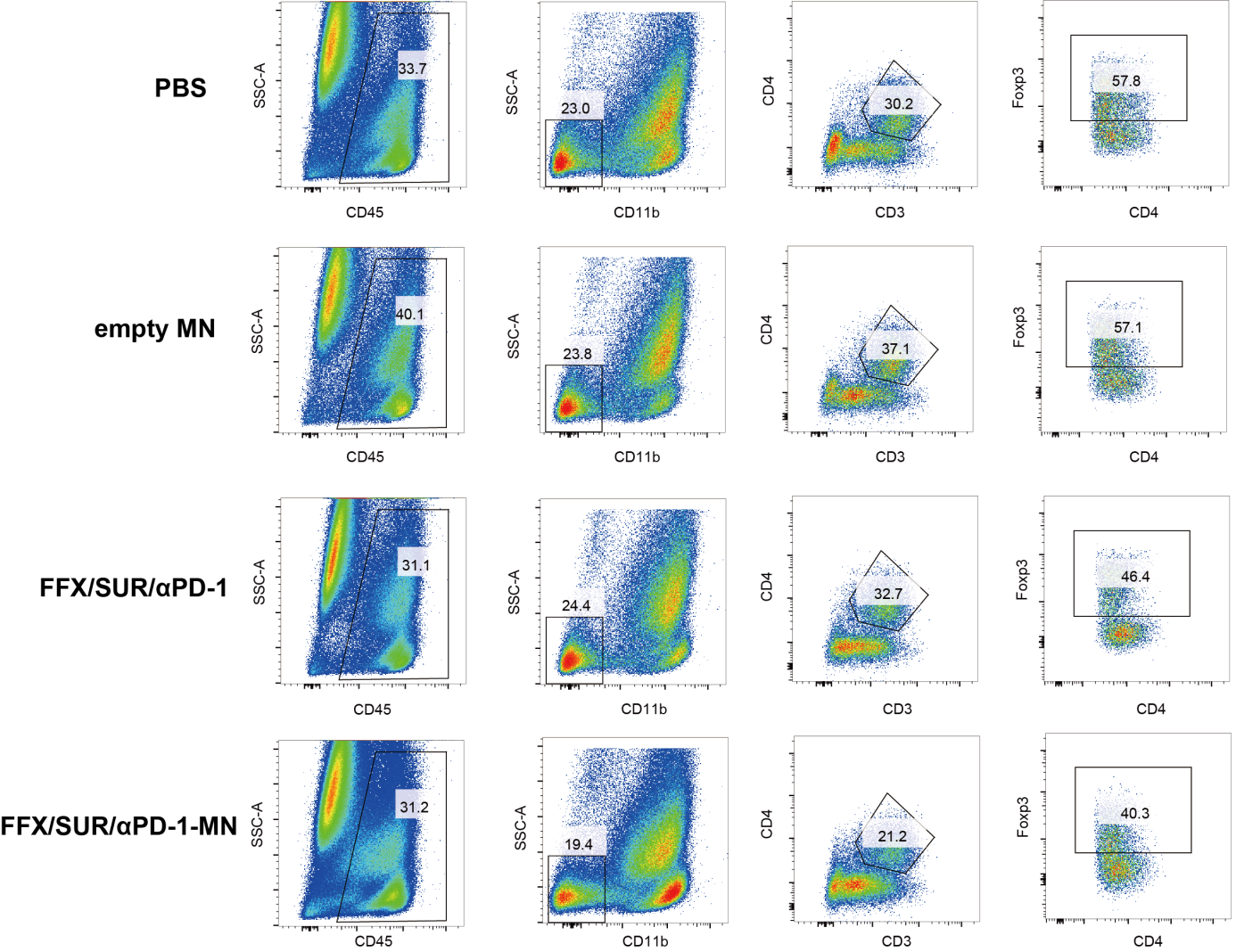


Figure S10. Flow cytometry gating strategy and representative plots showing the percentages of Tregs among CD45^+^ cells and among CD4^+^ T cells in the PDAC tumor microenvironment on day 21 after treatment, as shown in Figure 4D.


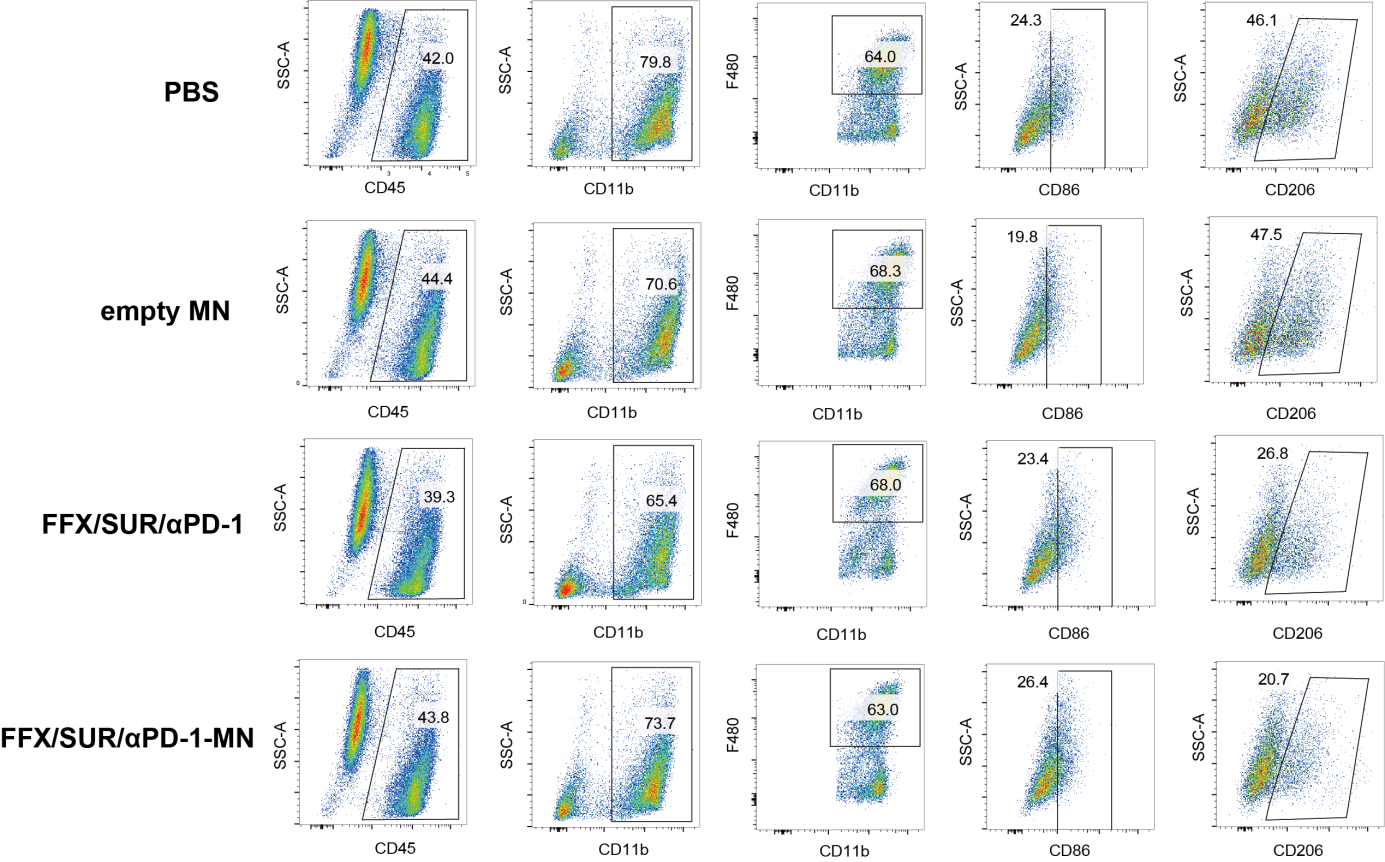


Figure S11. Flow cytometry gating strategy and representative plots showing the percentages of total macrophages, M1 macrophages, and M2 macrophages among CD45^+^ cells, and the percentage of M2 macrophages among total macrophages, in the PDAC tumor microenvironment on day 21 after treatment, as shown in Figure 4D.


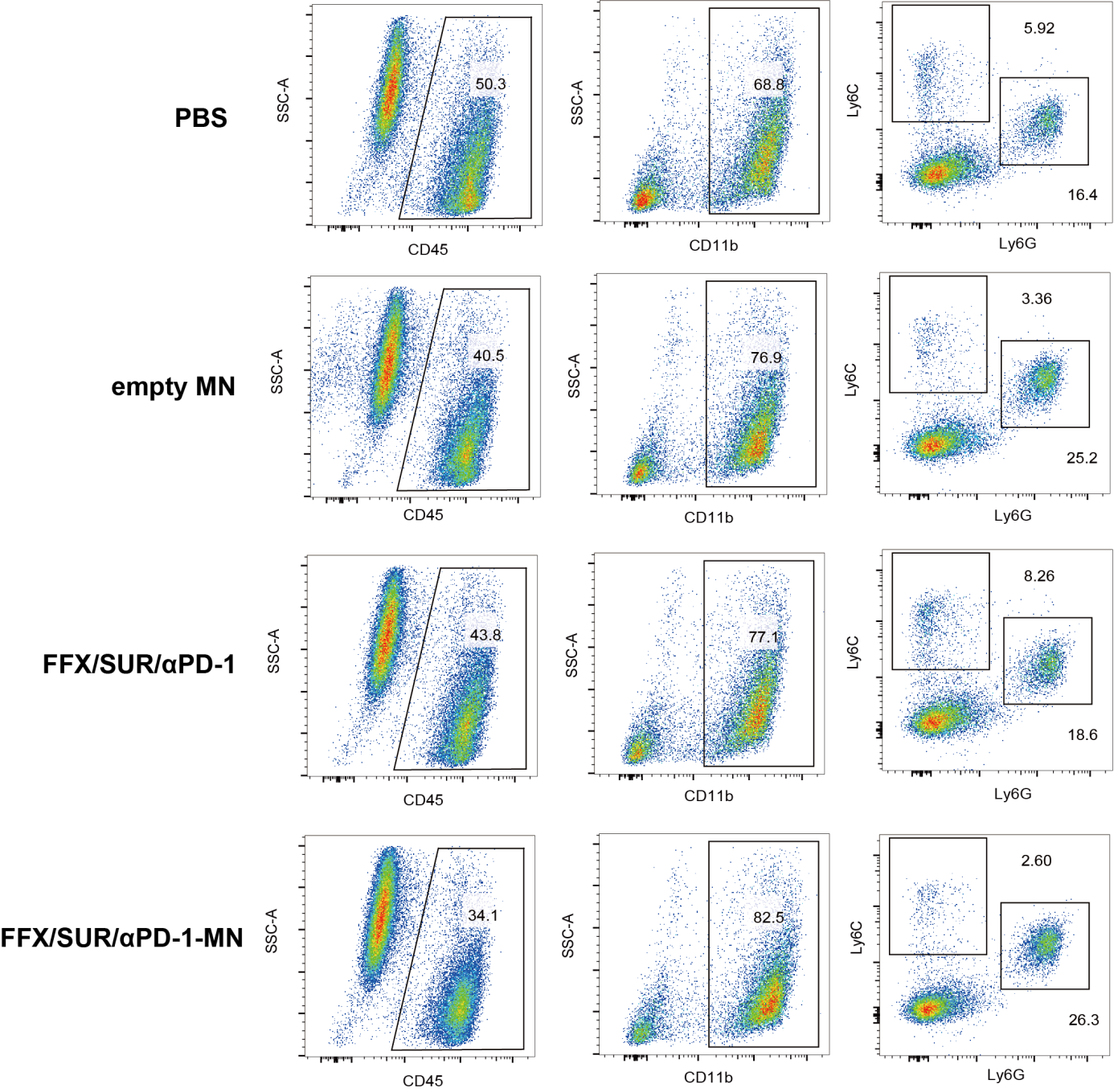


Figure S12. Flow cytometry gating strategy and representative plots showing the percentages of CD11b^+^ cells, PMN-MDSCs and M-MDSCs in CD45+ cells in the PDAC tumor microenvironment at day 21 after treatment, as shown in Figure 4D.


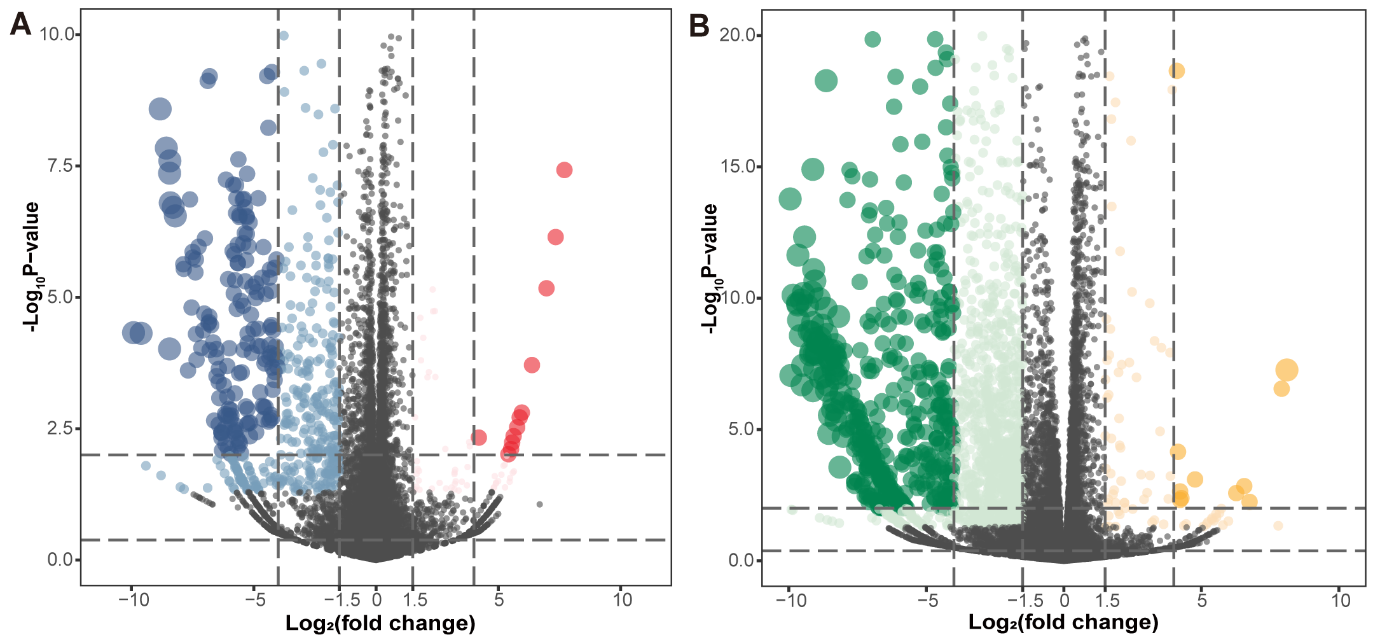


Figure S13. Volcano plots revealing treatment-specific gene expression changes in orthotopic Panc02 tumor-bearing mice. (A-B) Comparison of conventional systemic administration vs. PBS (A) and FFX/SUR/αPD-1-MN vs. empty MN (B) (n = 3, biologically independent samples).


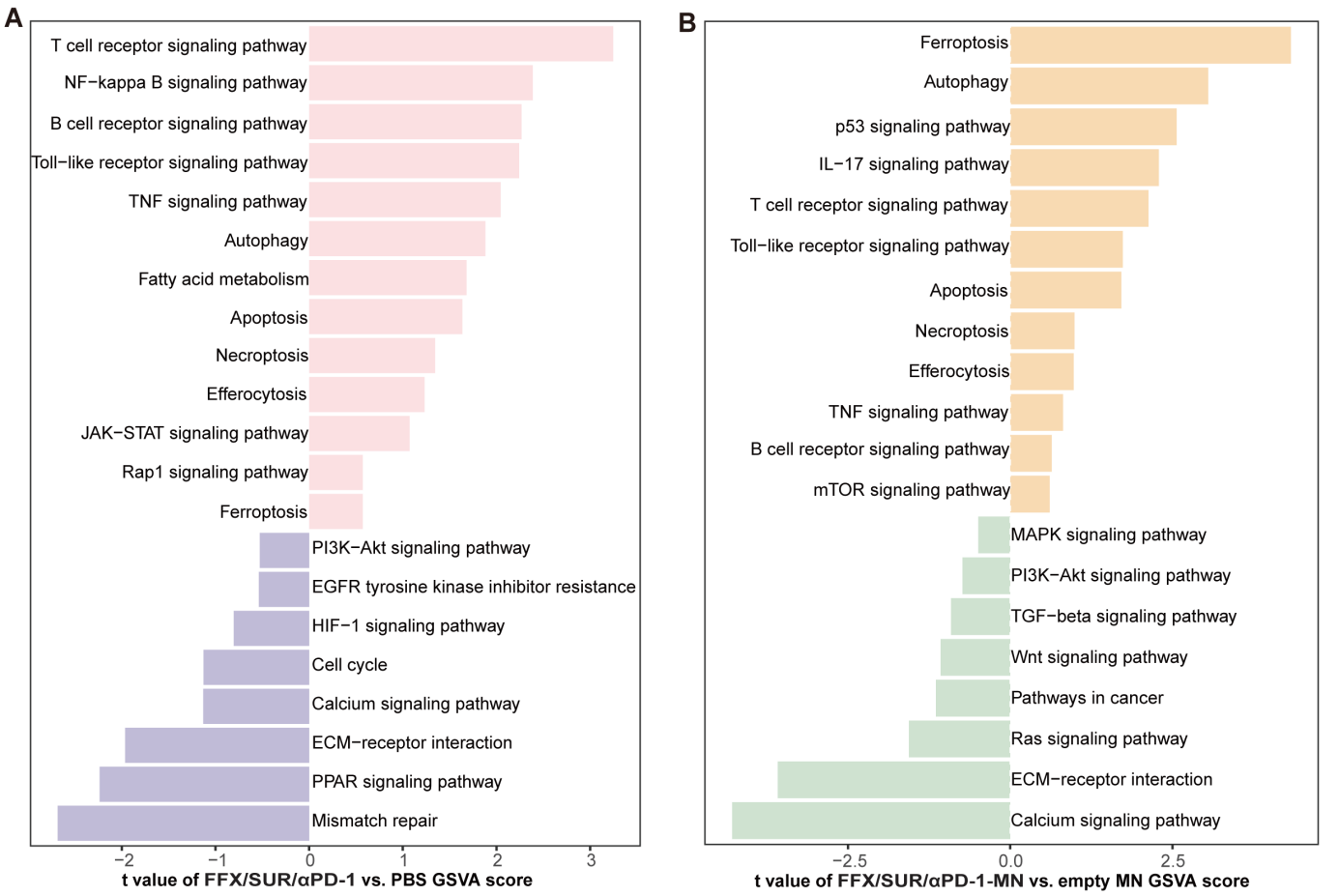


Figure S14. Comparative pathway enrichment analysis reveals differential modulation of tumor-associated pathways by MN-based vs. conventional drug delivery in orthotopic Panc02 tumor-bearing mice. (A-B) Comparison of conventional systemic administration vs. PBS (A) and FFX/SUR/αPD-1-MN vs. empty MN (B) (*n* = 3, biologically independent samples).


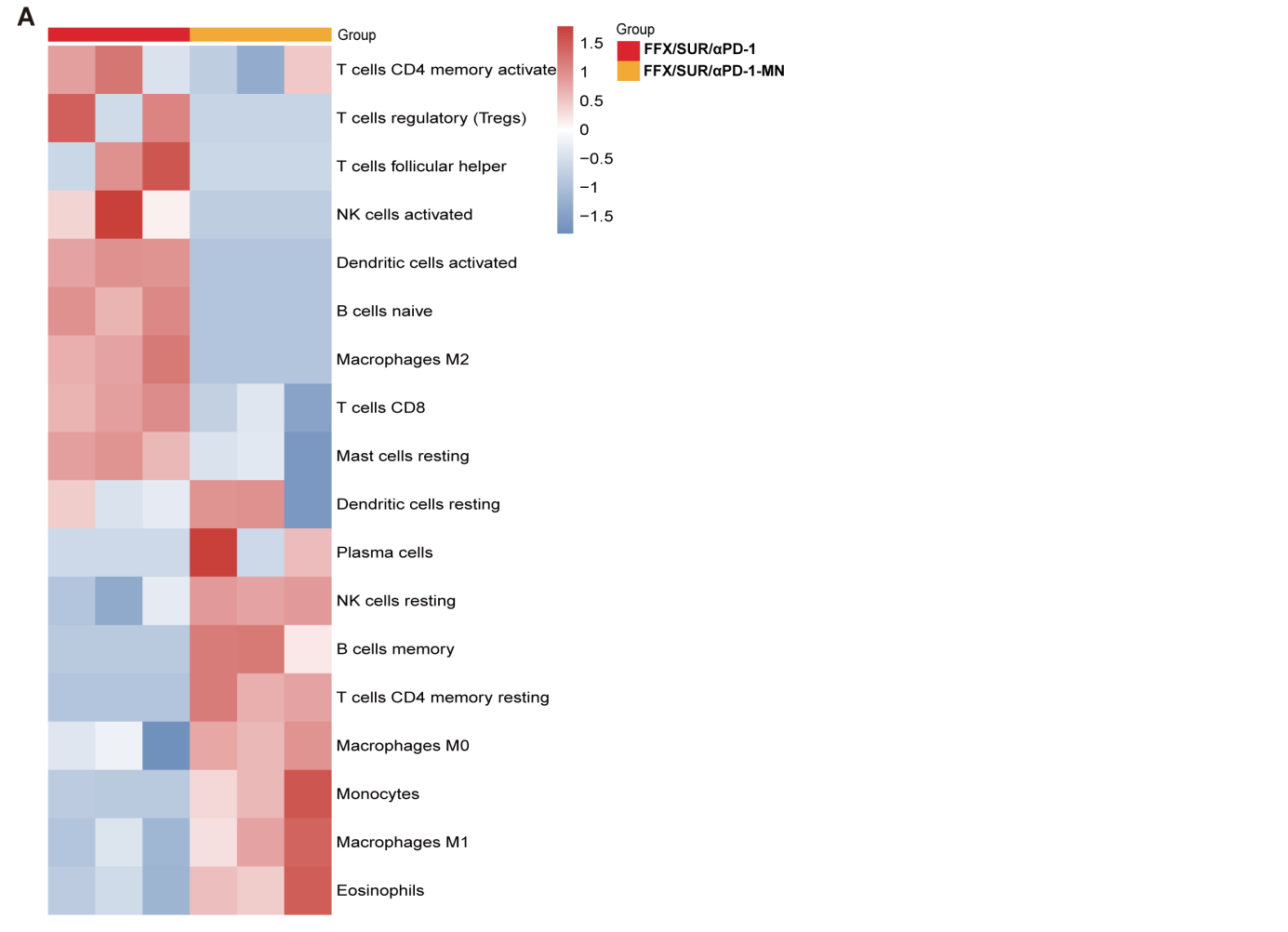


Figure S15. Comparative immune cell infiltration profiles in orthotopic Panc02 tumor-bearing mice, conventional systemic administration vs. FFX/SUR/αPD-1-MN (*n* = 3, biologically independent samples).


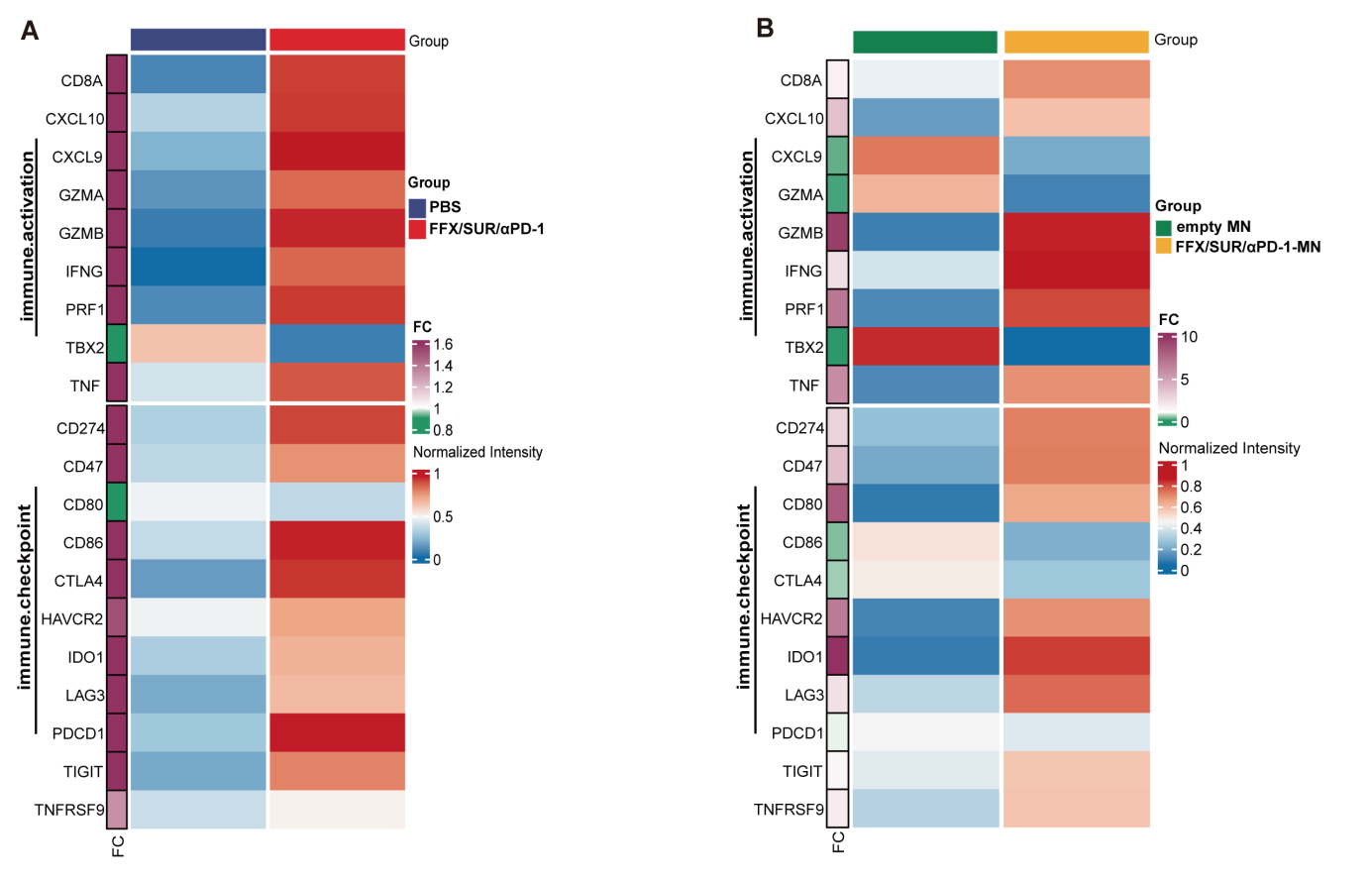


Figure S16. Comparative immune activation and checkpoint modulation in orthotopic Panc02 tumor-bearing mice. (A-B) Conventional systemic administration vs. PBS (A) and FFX/SUR/αPD-1-MN vs. empty MN (B) (*n* = 3, biologically independent samples).


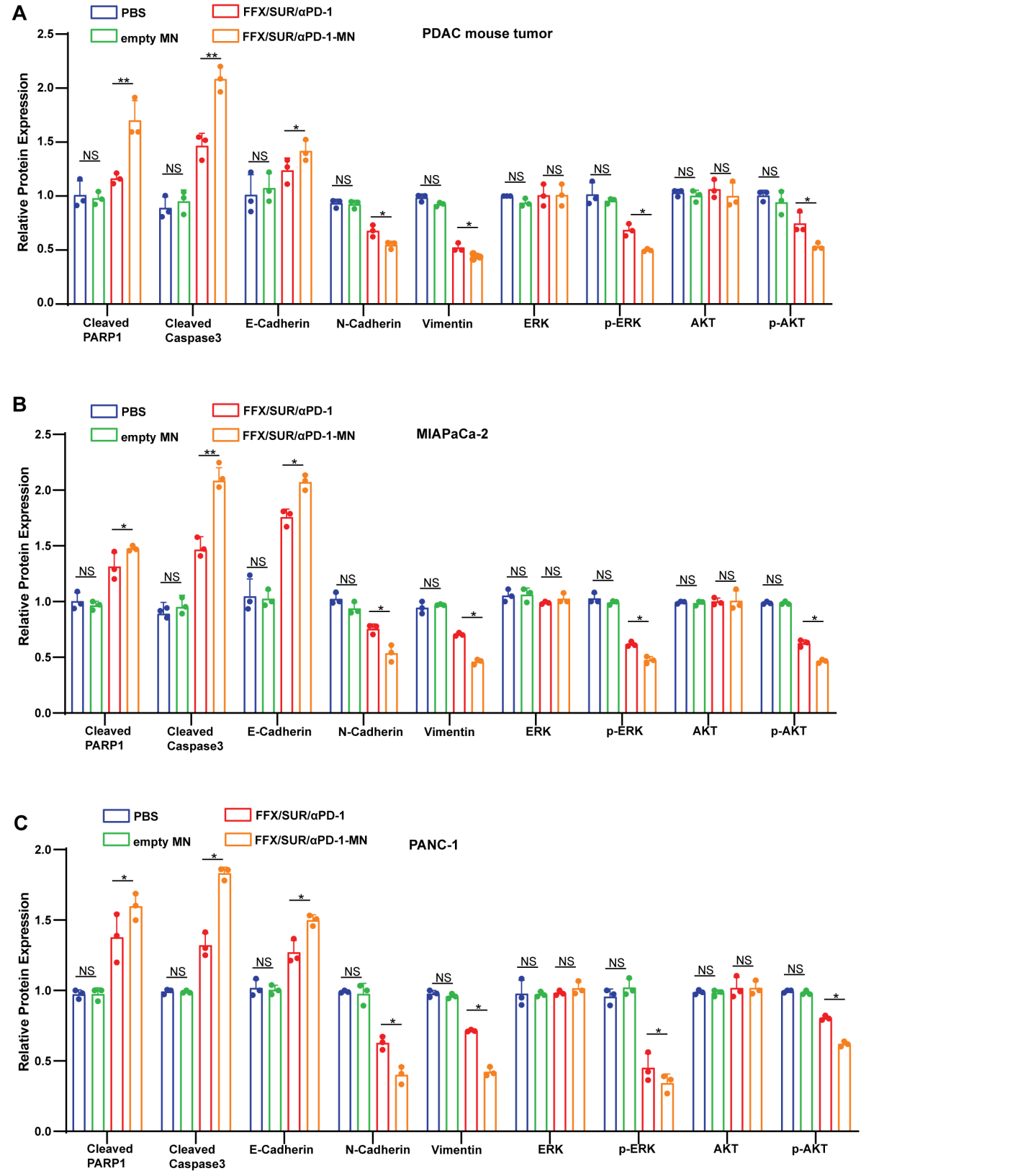


Figure S17. Quantification of Western blot protein expression in tumor tissues (A) and in human PDAC cell lines MIAPaCa-2 and PANC-1 (B-C) from different treatment groups (*n* = 3, biologically independent samples). Statistical analysis was performed using unpaired two-tailed Student’s *t*-test (A-C). Data are presented as the mean ± SD. ****P* < 0.001, ***P* < 0.01, **P* < 0.05; NS, not significant.
